# Nuclear–Electronic Orbital General Rate Theory: Predicting Hydrogen Kinetic Isotope Effects in the Deep Tunneling Regime

**DOI:** 10.1021/acs.jctc.6c01175

**Published:** 2026-08-21

**Authors:** Eno Paenurk, Jang Mok Yoo, Sharon Hammes-Schiffer

**Affiliations:** Department of Chemistry, 6740 Princeton University, Princeton, New Jersey 08544, United States

## Abstract

Hydrogen transfer is a critical component of many chemical and biological processes. The ratio of rate constants for hydrogen and deuterium transfer defines the H/D kinetic isotope effect (KIE), which is a powerful tool for elucidating hydrogen transfer mechanisms. Interpretation of experimental H/D KIEs relies on accurate and affordable computational methods. However, due to their light mass, hydrogen and deuterium can undergo tunneling, which is challenging to describe in multidimensional molecular systems. Herein, we introduce the nuclear–electronic orbital general rate theory (NEO-GRT), which enables the efficient prediction of H/D KIEs based on full-dimensional molecular quantum chemistry calculations. The NEO-GRT approach describes the hydrogen transfer rate constant with a general expression that spans the vibrationally adiabatic and nonadiabatic hydrogen tunneling regimes. The input quantities are computed using NEO density functional theory, which treats the transferring hydrogen or deuterium nucleus quantum mechanically on the same level as the electrons. We investigate two intramolecular proton transfer reactions in organic molecules at temperatures down to 50 K to evaluate the performance of NEO-GRT by comparison to transition state theory and ring-polymer instanton theory. The KIEs computed with NEO-GRT agree with those calculated using ring-polymer instanton theory for the full-dimensional molecular systems at the same level of electronic structure theory. This agreement indicates that NEO-GRT captures the deep hydrogen tunneling effects, in contrast to transition state theory, which neglects such effects. Given its relatively low computational cost, NEO-GRT is a promising approach for predicting H/D KIEs in large organic and organometallic systems.

## Graphical abstract

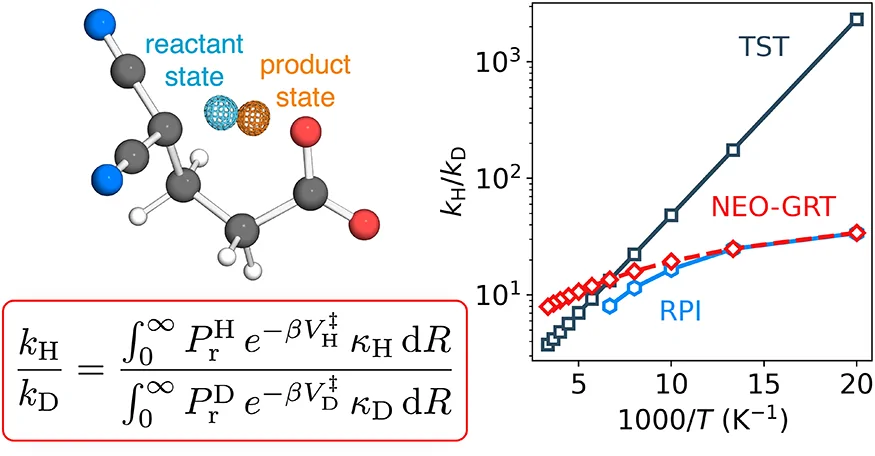

## 1 Introduction

Hydrogen transfer, which can occur as a proton, hydrogen atom, or hydride transfer, is a critical component of many chemical processes. Single-step or rate-determining hydrogen transfer reactions are often associated with significant primary H/D kinetic isotope effects (KIEs), defined as the ratio of rate constants for hydrogen and deuterium transfer

1 $$KIE=\frac{k_{H}}{k_{D}}$$

where *k*_L_ is the reaction rate constant for isotope L. H/D KIE measurements represent a powerful tool for investigating reaction mechanisms and can be used to interrogate individual reaction steps and catalytic cycles,^1–5^ as well as biological processes^6–11^ and proton-coupled electron transfer reactions.^12,13^ High H/D KIEs associated with significantly different reactivity for isotopically substituted positions have also been used to direct stereoselectivity^14,15^ and regioselectivity.^16^

KIEs are often explained in terms of transition state theory (TST). In TST, the KIE arises mainly from the difference in zero-point energies (ZPEs) of the isotopes^3^

2 $${KIE}_{TST}\approx \exp\left[-\beta \left(\Delta ZPE_{H}-\Delta ZPE_{D}\right)\right]$$

Here ΔZPE_L_ is the ZPE difference between the transition state and the reactant minimum for isotope L, and β = 1/*k*_B_ *T*, where *k*_B_ is the Boltzmann constant and *T* is the temperature. This approach has been estimated to give a maximum primary KIE at room temperature in the range of 7–10.^17^

In many cases, room-temperature H/D KIEs larger than 10 have been measured experimentally for proton transfer reactions,^18–21^ hydrogen atom transfer reactions^22–25^ and many organometallic reactions.^2,5,26,27^ These large KIEs implicate a significant degree of nuclear tunneling.^28–30^ These nuclear tunneling effects also influence the temperature dependence of KIEs. The TST formula in eq 2 predicts a linear plot for the logarithm of the KIE versus inverse temperature, whereas nuclear tunneling leads to a nonlinear plot that plateaus at low temperature.^22,30^

Several atomistic theories have been used to predict and explain KIEs for hydrogen tunneling reactions, such as instanton theory,^31–36^ ring-polymer molecular dynamics,^37–41^ the multiconfigurational time-dependent Hartree method,^42,43^ path integral free-energy perturbation theory,^44–48^ and semiclassical TST.^36,49–51^ The magnitudes and temperature dependences of the KIEs calculated with these methods can differ significantly from those calculated with TST^33,36^ and agree much better with experimental data.^32,42,43,49–51^ Although these methods capture nuclear tunneling effects and can achieve great accuracy, their computational cost is generally still too high to be routinely applicable to larger organic or organometallic systems that exhibit anomalously large KIEs.^2,5,25–27^ Various approximate multidimensional tunneling approaches have been developed within the TST framework^52,53^ and have been applied widely, including applications to enzymatic reactions.^54^ Despite the more approximate nature of these methods, they have often shown good accuracy in comparison to experiment and more sophisticated methods,^38,41,47,55–58^ although their accuracy can deteriorate at lower temperatures.^31,59^

An alternative approach for including nuclear quantum effects such as ZPE and tunneling is the nuclear–electronic orbital (NEO) method.^60–62^ The NEO method treats specified nuclei, typically hydrogen and deuterium, quantum mechanically on equal footing with electrons in a multicomponent quantum chemistry framework. NEO density functional theory (NEO-DFT)^63–65^ has proven to be particularly useful, as it efficiently treats electron–proton correlation (epc) with the epc functionals,^64–66^ enabling accurate calculations at a computational cost comparable to that of conventional electronic DFT. NEO-DFT has been shown to predict accurate gas-phase proton affinities^64,67^ and solution-phase p*K*_a_ values.^68^ The NEO multistate DFT (NEO-MSDFT) method can reproduce hydrogen tunneling splitting values in quantitative agreement with numerically exact grid-based calculations for fixed molecular geometries,^69,70^ and it has also been used for direct dynamics simulations of hydrogen tunneling.^71,72^ Although constrained NEO-DFT has been used to calculate rate constants in the TST framework for reactions with minimal tunneling,^73^ a NEO-based rate theory that would capture deep tunneling is desirable.

In this work, we present a general rate theory (GRT) for computing primary H/D KIEs with full-dimensional, atomistic NEO-DFT calculations. This approach treats the transferring hydrogen or deuterium quantum mechanically at the same level as the electrons and utilizes a general rate constant expression that includes nuclear tunneling and spans the vibrationally adiabatic and nonadiabatic regimes. We apply this NEO-GRT approach to the two intramolecular proton transfer reactions shown in Scheme 1. We use NEO-GRT to compute the KIE at different temperatures and compare the results to conventional TST and to ring-polymer instanton theory, which has been found to be accurate at low temperatures.^33^ We emphasize that the NEO-GRT, TST, and instanton theory calculations are all performed for the full-dimensional molecular systems using the same level of electronic structure theory to enable a direct comparison.

**Scheme 1:**
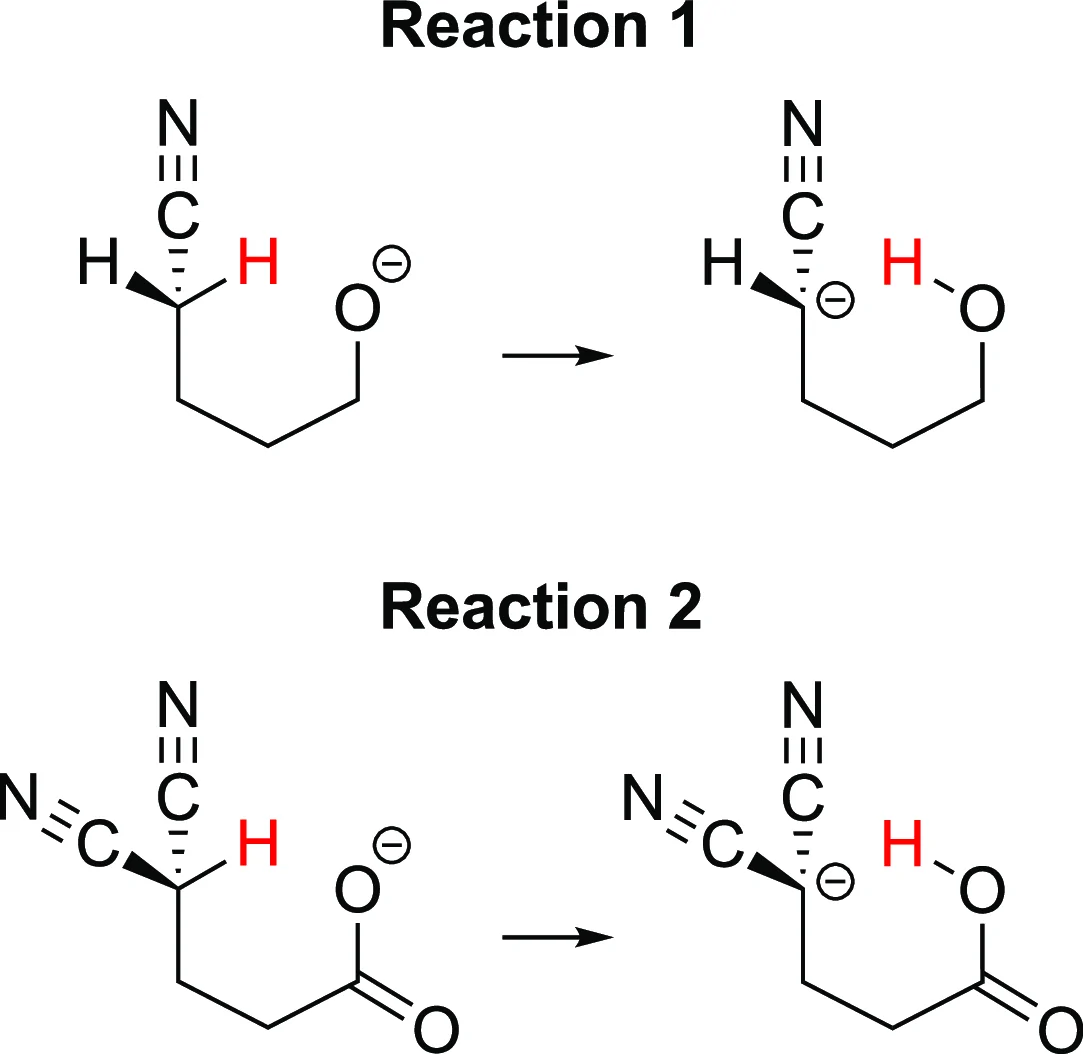
The Intramolecular Proton Transfer Reactions Studied in This Work^a^

a The transferring proton that was isotopically exchanged is shown in red.

This paper is organized as follows. Section 2 outlines the theoretical considerations and historical context of vibrationally nonadiabatic hydrogen tunneling rate theories, presents the GRT and KIE expression for hydrogen transfer, explains the relevant NEO-DFT methodology, and summarizes the computational workflow. Section 3 provides the computational details of the calculations performed in this paper. Section 4 presents the results and analysis, and Section 5 summarizes the work.

## 2 Theory

### 2.1 General Considerations

To calculate KIEs, we need to first formulate a general rate constant expression. We formulate our rate theory for describing hydrogen tunneling by treating the transferring hydrogen nucleus quantum mechanically. Here, we assume that the hydrogen transfer reaction is electronically adiabatic (i.e., remains on the electronic ground state) but could be vibrationally adiabatic or nonadiabatic (i.e., involves excited proton vibrational states).^74–76^ The extension to electronically nonadiabatic systems is relevant to proton-coupled electron transfer (PCET)^77^ and will be the subject of future work.

A schematic description of electronically adiabatic hydrogen tunneling is shown in Figure 1. We consider two diabatic vibronic states: the reactant state with the proton vibrational wave function localized near the proton donor (blue vibronic surface and proton vibrational wave functions) and the product state with the proton vibrational wave function localized near the proton acceptor (orange vibronic surface and proton vibrational wave functions). We use the general term diabatic vibronic states rather than diabatic vibrational states because each diabatic state is the product of the ground electronic state and a proton vibrational state. Hydrogen tunneling is described by a transition from the reactant vibronic state to the product vibronic state. For this transition to conserve energy, the two diabatic vibronic states must have equal energy (*V*_*a*_ = *V*_*b*_). The lowest-energy geometry at which this condition is satisfied is called the minimum energy crossing point (MECP). For hydrogen tunneling systems, at the MECP the adiabatic proton vibrational wave functions are delocalized in a symmetric or nearly symmetric double-well potential (green and gray proton vibrational wave functions), and the lowest two adiabatic proton vibrational states are split by the tunneling splitting Δ, which is twice the vibronic coupling (Δ = 2|*V*_*ab*_|). This type of MECP is also sometimes referred to as the tunneling-ready state,^10,78–80^ emphasizing that it is the heavy-atom configuration at which hydrogen tunneling can occur. In this work, the heavy-atom configuration refers to the configuration of all nuclei excluding the transferring hydrogen.

**Figure 1:**
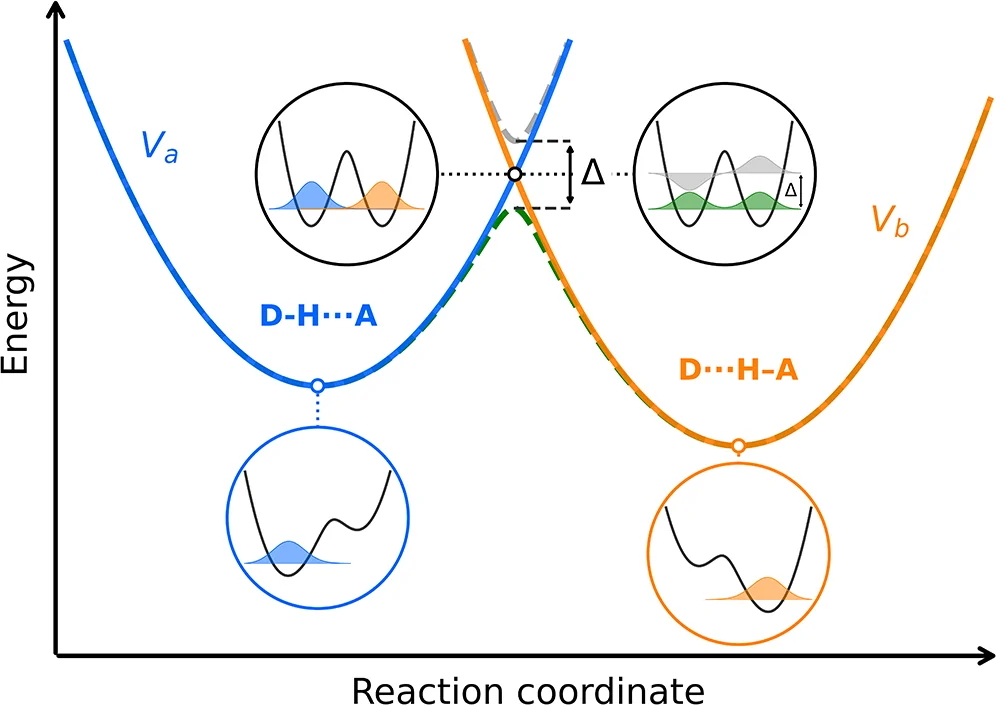
Schematic illustration of electronically adiabatic hydrogen tunneling. The one-dimensional vibronic surfaces are plotted as a function of the reaction coordinate, which is composed of motions from all nuclei other than the transferring hydrogen. The donor–acceptor distance is often treated explicitly and therefore also excluded from this reaction coordinate. The reactant (blue) and product (orange) diabatic vibronic surfaces correspond to the transferring proton vibrational wave function localized near the donor or acceptor, respectively. The diabatic vibronic surfaces cross at the minimum energy crossing point (MECP, denoted with a black circle), where the adiabatic proton vibrational states are split by the tunneling splitting Δ. The insets at marked positions along the reaction coordinate show the electronically adiabatic proton potential energy profile as a function of the transferring hydrogen position coordinate in black, together with the proton vibrational wave functions associated with the diabatic proton vibrational states in blue and orange or the adiabatic proton vibrational states in green and gray. Note that the adiabatic and diabatic proton vibrational states are equivalent to each other at the minima of the diabatic vibronic surfaces but differ at the MECP.

In this formulation, the reaction coordinate does not include the transferring hydrogen, which is treated quantum mechanically. Thus, the reaction coordinate is composed of only the other nuclear coordinates, defined as heavy atoms herein. Moreover, as discussed below, the proton donor–acceptor distance *R* is treated explicitly through a thermal averaging procedure and therefore also does not contribute to the reaction coordinate.

A general rate theory must account for the following characteristics of hydrogen transfer reactions:

1. The reaction can be vibrationally adiabatic or nonadiabatic or in the intermediate regime, depending on the magnitude of the vibronic coupling. The reaction is vibrationally adiabatic if |*V*_*ab*_| ≫ *k*_B_ *T* and is vibrationally nonadiabatic if |*V*_*ab*_| ≪ *k*_B_ *T*.
2. The vibronic coupling is strongly dependent on the proton donor–acceptor distance: the vibronic coupling is larger at shorter distances and smaller at longer distances.^81–83^

3. The proton donor–acceptor distance motion is usually a soft vibrational mode, and therefore a wide range of distances may be sampled.

The third point implies that we should calculate a thermal average of the rate constant over a range of different proton donor–acceptor distances. This procedure was also used in earlier studies of hydrogen transfer.^84,85^ The donor–acceptor distance dependence is also commonly included in studies of biological hydrogen transfer^86,87^ and PCET.^13,77,88^ The second and third points suggest that the reaction could change from vibrationally nonadiabatic to vibrationally adiabatic when shorter donor–acceptor distances are thermally accessible. The rate constant expression should thus handle both regimes as well as the intermediate regime.

The rate constant for a vibrationally nonadiabatic reaction can be expressed in terms of Fermi’s golden rule. A variety of golden rule-based calculations of proton transfer have been reported.^81,82,84,89–92^ Proton transfer rate theories that account for both vibrationally adiabatic and nonadiabatic regimes via the Landau–Zener (LZ)^93,94^ formalism have also been developed.^83,95–101^ These theories have been applied to low-dimensional model potentials, which have provided fundamental insights into proton transfer kinetics. However, multidimensional atomistic methods are desirable for modeling and understanding proton transfer reactions in realistic molecular systems.^102^ In this paper, we develop such an atomistic approach using NEO-DFT to quantize the transferring hydrogen or deuterium nucleus.

For the formalism presented herein, we only account for tunneling from the reactant diabatic ground vibronic state to the product diabatic ground vibronic state. It has been shown that especially for deuterium, excited vibrational states might be required for reliable accuracy at room temperature.^91,98,101,103,104^ We are currently expanding this theory to include excited vibrational states, but here we focus on low-temperature reactions where such contributions are not significant.

### 2.2 Rate Constant Expression

This subsection presents the rate constant expressions that form the basis of our theory for describing hydrogen transfer reactions. The transferring hydrogen or deuterium nucleus is treated quantum mechanically via the NEO approach, and the reaction is described in terms of reactant and product diabatic vibronic states. As mentioned above, the vibronic coupling between these diabatic vibronic states depends strongly on the proton donor–acceptor distance *R*. As also mentioned above, the proton donor–acceptor distance *R* is treated explicitly through a thermal averaging procedure and hence does not contribute to the reaction coordinate. In principle, all quantities in the rate constant expressions depend on *R*, although in some cases this dependence is sufficiently weak that it can be neglected.

We express the canonical rate constant at temperature *T* as^84^

3 $$k\left(T\right)=\int_{0}^{\infty }P_{r}\left(T,R\right)k\left(T,R\right)dR$$

where *P*_r_(*T*, *R*) is the Boltzmann probability for the reactant, and *k*(*T*, *R*) is the canonical rate constant at temperature *T* and donor–acceptor distance *R*. The Boltzmann probability for the reactant is

4 $$P_{r}\left(T,R\right)=\frac{e^{-\beta V_{r}\left(R\right)}Q_{r}\left(T,R\right)}{\int_{0}^{\infty }e^{-\beta V_{r}\left(R^{\prime }\right)}Q_{r}\left(T,R^{\prime }\right)dR^{\prime }}\approx \frac{e^{-\beta V_{r}\left(R\right)}}{\int_{0}^{\infty }e^{-\beta V_{r}\left(R^{\prime }\right)}dR^{\prime }}$$

where *V*_r_(*R*) is the energy of the reactant with donor–acceptor distance *R* and *Q*_r_(*T*, *R*) is the reactant partition function at a fixed *R* value and temperature *T*. In this work, we approximate *Q*_r_(*R*, *T*) to be independent of *R* to simplify the definition of the Boltzmann probability for the reactant. In relation to Figure 1, *V*_r_(*R*) corresponds to the minimum of *V*_*a*_ for donor–acceptor distance *R*. In general, the curves in Figure 1 are distinct for each fixed *R* value.

For the rate constant *k*(*T*, *R*), we employ what is often called the nonadiabatic statistical theory (NAST) formalism.^105–108^ Including the explicit dependence on *R*, the NAST rate constant is

$$k\left(T,R\right)=\frac{1}{\beta h}\frac{Q^{‡}\left(T,R\right)}{Q_{r}\left(T,R\right)}\times e^{-\beta V^{‡}\left(R\right)}\times \kappa \left(T,R\right)$$ 5

where the first term contains the partition functions of the MECP (*Q* ^‡^) and the reactant (*Q*_r_), and *h* is Planck’s constant. Note again that the partition functions here correspond to a fixed *R* value. The second term is the thermal activation factor, where *V* ^‡^(*R*) is the activation energy defined as *V* ^‡^(*R*) = *V*_MECP_(*R*) – *V*_r_(*R*). The last term is the nonadiabatic transmission coefficient, defined as

6 $$\kappa \left(T,R\right)=\beta \int_{-V^{‡}\left(R\right)}^{\infty }P_{trans}\left(E,R\right)e^{-\beta E}dE$$

where *P*_trans_(*E*, *R*) is the nonadiabatic transmission probability at energy *E* relative to *V*_MECP_(*R*). See Section S1 for the derivation of this form of κ­(*T*, *R*). In the classical adiabatic limit, κ­(*T*, *R*) = 1, and eq 5 becomes the standard TST expression. According to our choice of the zero of energy, *E* < 0 corresponds to the energy associated with the reaction coordinate being less than the barrier height (see Figure S1). Thus, a nonzero value of *P*_trans_(*E*, *R*) for *E* < 0 corresponds to tunneling through the barrier along the reaction coordinate. Because this rate theory is formulated in terms of the diabatic vibronic states, where the reaction coordinate excludes the transferring, quantized proton, the tunneling through the barrier along the reaction coordinate captured by *P*_trans_(*E*, *R*) for *E* < 0 corresponds to tunneling of the nonquantized nuclei. Note that the tunneling of the transferring hydrogen or deuterium nucleus is included in *P*_trans_(*E*, *R*) via the NEO formalism for all energies *E*.

The transmission probability *P*_trans_(*E*, *R*) can be computed using the Landau–Zener (LZ) formula. Substituting $E=\frac{1}{2}\mu \left(R\right)v^{2}$ into eq 6 yields the velocity-averaged transmission coefficient based on Kuki’s distribution^108,109^

$$\kappa^{LZ}\left(T,R\right)=\beta \mu \left(R\right)\int_{0}^{\infty }vP_{trans}^{LZ}\left(v,R\right)e^{-\beta \mu \left(R\right)v^{2}/2}dv$$ 7

where the integral is over velocities *v* along the reaction coordinate with reduced mass μ­(*R*). See Section S1 for the derivation of this expression. The LZ formula is defined only for *E* > 0 and therefore does not account for quantum tunneling through the barrier associated with the nonquantized nuclei. Here, *P*_trans_^LZ^(*v*,*R*) is computed using the Holstein formula, which accounts for recrossings in the normal crossing regime^110,111^

8 $$P_{trans}^{LZ}\left(v,R\right)=\frac{2P^{LZ}\left(v,R\right)}{1+P^{LZ}\left(v,R\right)}$$

where *P* ^LZ^(*v*, *R*) is the LZ transition probability for a single crossing event^93,94^

9 $$P^{LZ}\left(v,R\right)=1-\exp\left[-\frac{2\pi |V_{ab}\left(R\right){|}^{2}}{\hbar v\left|\Delta g\left(R\right)\right|}\right]$$

In this expression, |*V*_*ab*_(*R*)| is the vibronic coupling at the MECP, and Δ ***g*** (*R*) = ***g***_*a*_(*R*) – ***g***_*b*_(*R*), where ***g***_*n*_(*R*) is the gradient on the diabatic vibronic surface *n* at the MECP.

To account for quantum tunneling effects associated with the nonquantized nuclei, the weak coupling (WC) probability expression^108,112^ can be employed. The WC transmission probability is of the form

10 $$P_{trans}^{WC}\left(E,R\right)=4\pi^{2}|V_{ab}\left(R\right){|}^{2}{\left(\frac{2\mu \left(R\right)}{\hbar^{2}\mathrm{g̅}\left(R\right)\left|\Delta g\left(R\right)\right|}\right)}^{2/3}\times {Ai}^{2}\left(-E{\left(\frac{2\mu \left(R\right){\left|\Delta g\left(R\right)\right|}^{2}}{\hbar^{2}\mathrm{g̅}{\left(R\right)}^{4}}\right)}^{1/3}\right)$$

Here Ai­(*x*) is the Airy function, and $\mathrm{g̅}\left(R\right)={\left(|g_{a}\left(R\right)∥g_{b}\left(R\right)|\right)}^{1/2}$ . Equation 10 is substituted into eq 6 to obtain κ^WC^(*T, R*). A known limitation of the WC formula is that it can predict values of *P*_trans_^WC^(*E*,*R*) greater than unity for strong vibronic coupling, large reduced masses, or small gradients.^107^ In such cases, we set *P*_trans_^WC^(*E*,*R*) to unity. For the two reactions studied herein, this behavior was observed for only one case, specifically H transfer in Reaction 2 for a donor–acceptor distance range of 0.04 Å, and thus was not relevant.

We emphasize that the tunneling of the transferring hydrogen or deuterium nucleus is included in both the LZ and WC transmission probabilities. Specifically, the square of the vibronic coupling, |*V*_*ab*_(*R*)|^2^, is related to the probability of hydrogen or deuterium tunneling. In our implementation, this vibronic coupling is computed at the MECP using NEO-MSDFT, as discussed further below.

To calculate the rate constant with this formalism, using either the LZ or the WC transmission probability, we need to compute the following quantities: μ­(*R*), ***g***_*a*_(*R*), ***g***_*b*_(*R*), *V*_r_(*R*), *V*_MECP_(*R*), and |*V*_*ab*_(*R*)|. The methods used to compute these quantities are described below.

### 2.3 KIE Expression

For the KIE calculations, we assume that the partition function ratios in eq 5 largely cancel between the two isotopes. This assumption is justified because the translational and rotational partition function ratios are similar for the two isotopes. Moreover, the transferring hydrogen or deuterium is treated quantum mechanically within the NEO framework, and therefore its vibrational states do not contribute to the vibrational partition function. Thus, the isotope effect on the vibrational partition function is expected to be small as well. The KIE can then be expressed as

$$\frac{k_{H}\left(T\right)}{k_{D}\left(T\right)}=\frac{\int_{0}^{\infty }P_{r}^{H}\left(T,R\right)e^{-\beta V_{H}^{‡}\left(R\right)}\kappa_{H}\left(T,R\right)dR}{\int_{0}^{\infty }P_{r}^{D}\left(T,R\right)e^{-\beta V_{D}^{‡}\left(R\right)}\kappa_{D}\left(T,R\right)dR}$$ 11

We used eq 11 for all KIE calculations in this work.

### 2.4 Nuclear–Electronic Orbital Theory

We quantize the transferring hydrogen or deuterium nucleus using NEO-DFT,^63–65^ which incorporates electron exchange–correlation with conventional electronic functionals and electron–proton correlation with the epc functionals.^64–66^ The description of delocalized, bilobal protonic densities characteristic of hydrogen tunneling requires a multireference treatment within the NEO framework.^69,113,114^ Consider a proton moving in a symmetric double-well potential in the conventional Born–Oppenheimer picture. Within the NEO framework, the quantum proton in such cases is represented by two distinct basis function centers, each one located approximately in the donor or acceptor well. Even with this flexibility, however, the variational NEO-DFT solution for the proton density using the available epc functionals will localize in either the donor or acceptor well^69,70^ due to insufficient electron–proton correlation.^113^ This feature enables us to use NEO-DFT to define the diabatic vibronic surfaces in terms of localized proton densities even at the MECP, which typically corresponds to a symmetric or nearly symmetric double-well potential (Figure 1).

The vibronic coupling values at the MECP can be calculated with NEO multistate DFT (NEO-MSDFT),^69,70^ which combines the localized NEO-DFT states with a nonorthogonal configuration interaction scheme.^115^ For symmetric or nearly symmetric environments, the adiabatic NEO-MSDFT vibronic states correspond to delocalized, bilobal proton densities, as shown in Figure 2 for malonaldehyde. In this case, the difference between the energies of the two adiabatic NEO-MSDFT vibronic states is the tunneling splitting. The vibronic coupling between the two symmetrically orthogonalized localized NEO-DFT vibronic states is

12 $$V_{ab}=\frac{1}{1-S_{ab}^{2}}\left|H_{ab}-\frac{H_{a}+H_{b}}{2}S_{ab}\right|$$

where *S*_*ab*_ is the overlap between the nonorthogonal localized vibronic states. Here, *H*_*a*_ and *H*_*b*_ are the diagonal elements and *H*_*ab*_ is the off-diagonal element of the effective NEO-MSDFT Hamiltonian.^69^ The diagonal elements are simply the respective NEO-DFT energies, while the off-diagonal element is represented by a physically motivated form inspired by conventional electronic MSDFT.^116–118^ When *H*_*a*_ = *H*_*b*_, the tunneling splitting is 2|*V*_*ab*_|.

**Figure 2:**
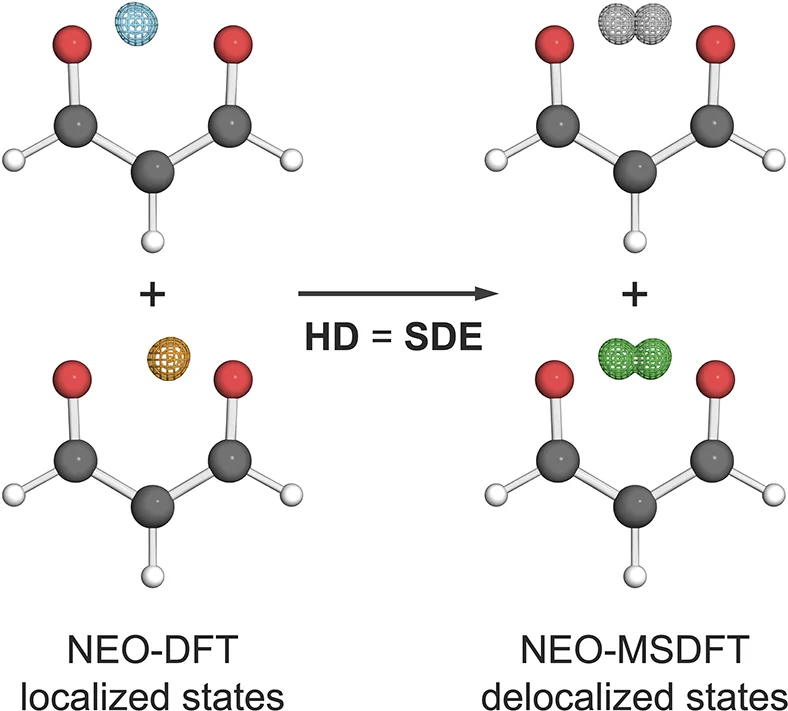
Visual representation of the NEO-MSDFT procedure for malonaldehyde. Two localized NEO-DFT diabatic vibronic states are combined to produce delocalized NEO-MSDFT adiabatic ground and first excited vibronic states. The proton densities are shown with an isovalue of 0.01 au using a color scheme that is consistent with Figure 1. Although the phase difference in the adiabatic first excited vibronic state wave function is not visible in the proton density (shown in gray, top right), the corresponding node creates a sharper boundary between the two lobes of the proton density.

The tunneling splittings are sensitive to small errors in the proton densities arising from approximations underlying the electron–proton correlation functional and the form of the off-diagonal elements. In previous work, the *S*_*ab*_ overlap element in NEO-MSDFT was scaled according to a functional form with two parameters that were fit to reproduce numerically exact reference tunneling splittings for a small model system.^69^ We found this scaling scheme to be less accurate at short donor–acceptor distances and high coupling values. Thus, we derived an alternative scaling scheme that performs better across the entire range of donor–acceptor distances necessary for rate constant and KIE calculations. This scaling scheme corresponds to

13 $$S_{ab}^{\prime }=S_{ab}^{\beta }$$

14 $$V_{ab}^{\prime }=\frac{\alpha }{1-{\left(S_{ab}^{\prime }\right)}^{2}}\left|H_{ab}-\frac{H_{a}+H_{b}}{2}S_{ab}^{\prime }\right|$$

Here, α and β are parameters to be optimized for small model systems and subsequently held fixed. The new and original scaling schemes agree well for relatively small overlap values at longer donor–acceptor distances but differ for larger overlap values at smaller donor–acceptor distances. The justification of this form, its parametrization, and a detailed analysis are provided in Section S3. We emphasize that the two scaling parameters are determined by reproducing numerically exact reference tunneling splittings for small model systems and then remain fixed for all future applications of NEO-MSDFT, analogous to the parameters in density functionals.

### 2.5 NEO Minimum Energy Crossing Point

Our rate theory requires finding the MECP geometry for the NEO-DFT vibronic energy surfaces and calculating the vibronic coupling at that geometry. Within the NEO framework, the MECP is defined as a geometry of the classical nuclear coordinates **R**_c_, for which there are two localized NEO-DFT solutions with equal energies and distinct quantum nuclear position expectation values (i.e., ⟨**r**⟩_*a*_ ≠ ⟨**r**⟩_*b*_, where **r** is the proton coordinate and the *a* and *b* subscripts of the brackets indicate the reactant and product diabatic vibronic states, respectively). For hydrogen transfer reactions, this inequality signifies that one quantum nuclear position expectation value is near the donor and the other is near the acceptor.

For finite basis sets, the NEO-DFT energy depends not only on the classical nuclear coordinates, but also on the quantum nuclear basis function center coordinates. Typically the position of the quantum nuclear basis function center is optimized variationally in conjunction with the self-consistent field procedure of NEO-DFT. For practical purposes, we define the diabatic states in terms of distinct nuclear basis function centers, i.e., one on the donor side, $R_{a}$ , and one on the acceptor side, $R_{b}$ . We also express the NEO-DFT energy as a function of the nuclear basis function center position as well as the classical nuclear coordinates. Thus, our two diabatic vibronic surfaces are $V_{a}\equiv E^{NEO}\left(R_{a},R_{c}\right)$ and $V_{b}\equiv E^{NEO}\left(R_{b},R_{c}\right)$ , where $E^{NEO}\left(R_{n},R_{c}\right)$ is the NEO-DFT energy at the classical nuclear coordinates **R**_c_ with the quantum nuclear basis function center at $R_{n}$ . Our goal is to find coordinates $R_{a}$ , $R_{b}$ , and **R**_c_ that minimize $E^{NEO}\left(R_{a},R_{c}\right)$ (or $E^{NEO}\left(R_{b},R_{c}\right)$ ) such that $E^{NEO}\left(R_{a},R_{c}\right)=E^{NEO}\left(R_{b},R_{c}\right)$ and ⟨**r**⟩_*a*_ ≠ ⟨**r**⟩_*b*_. It is important to note that at the MECP, the quantum nucleus should be in the vibronic ground state. In other words, although the MECP is generally not an energy minimum of either diabatic vibronic surface with respect to the classical nuclear coordinates, the quantum proton basis function center should be relaxed to its minimum on its respective diabatic vibronic surface.

We use the effective gradient approach by Harvey^119^ to locate the MECP, although other methods could be implemented instead.^120,121^ We define a gradient orthogonal to the crossing seam of the two diabatic vibronic surfaces

15 $$g_{⊥}=g_{a}-g_{b}$$

and a gradient parallel to the crossing seam

16 $$g_{∥}=g_{a}-\frac{g_{⊥}}{\left|g_{⊥}\right|}\left(g_{a}\cdot \frac{g_{⊥}}{\left|g_{⊥}\right|}\right)$$

We then define an effective gradient pointing toward the MECP as

17 $$g_{eff}=\alpha_{MECP}\left(V_{a}-V_{b}\right)\frac{g_{⊥}}{\left|g_{⊥}\right|}+g_{∥}$$

where the gradient term pointing toward the crossing seam is normalized, as suggested by Schlegel and co-workers,^122^ and scaled by the energy difference between the diabats and a scaling parameter α_MECP_, which has been used with values up to 30 *E*_h_^–1^.^123^ Scaling by α_MECP_ allows us to tune how stringently we want to converge the energy difference between the diabatic surfaces compared to the convergence to the minimum of the crossing seam.

To optimize the NEO MECP as defined above, we use eq 17 as the gradients of the classical nuclear coordinates, while maintaining the respective diabatic vibronic state gradients on the quantum nuclear basis function centers, such that the resulting NEO effective gradient is

18 $$g_{eff}^{NEO}=\left(\begin{array}{l} g_{a}^{\left(R_{a}\right)}\\ g_{b}^{\left(R_{b}\right)}\\ g_{eff}^{\left(R_{c}\right)} \end{array}\right)$$

Note that $g_{a}^{\left(R_{a}\right)}=\partial V_{a}/\partial R_{a}$ and $g_{b}^{\left(R_{b}\right)}=\partial V_{b}/\partial R_{b}$ are three-dimensional vectors, and $g_{eff}^{\left(R_{c}\right)}$ is a 3*N*_c_-dimensional vector obtained using eqs 15–17, where ***g***_*a*_ ≡ ***g***_*a*_^(**R**_c_)^ = ∇_**R**_c__ *V*_*a*_ and ***g***_*b*_ ≡ ***g***_*b*_^(**R**_c_)^ = ∇_**R**_c__ *V*_*b*_. Minimizing along this gradient leads to the desired NEO MECP as defined above.

### 2.6 Input Quantities for Rate Constant and KIE

To calculate the quantities required to compute the KIE, we use the gradients and reduced Hessian matrix elements with respect to the classical nuclear coordinates, assuming the nuclear basis function centers respond instantaneously to the motion of the classical nuclei. The gradient and reduced Hessian are defined as

19 $$g_{n}\equiv g_{n}^{\left(R_{c}\right)}$$

20 $$H_{n}={\left[H_{n}^{ext}\right]}_{cc}-{\left[H_{n}^{ext}\right]}_{qc}^{T}{\left[H_{n}^{ext}\right]}_{qq}^{-1}{\left[H_{n}^{ext}\right]}_{qc}$$

Here, **H**_*n*_^ext^ is the extended 3­(*N*_c_ + 1) × 3­(*N*_c_ + 1) Hessian matrix corresponding to second derivatives of the diabatic vibronic surface *n* with respect to the classical nuclear coordinates and the respective nuclear basis function center $R_{n}$ . The reduced Hessian ***H***_*n*_ is a 3*N*_c_ × 3*N*_c_ matrix defined such that the nuclear basis function center responds instantaneously to the motion of the classical nuclear coordinates. For the submatrices, the subscripts “c” and “q” denote the classical nuclear coordinates and quantum nuclear basis function center coordinates, respectively. These submatrices are defined as ${\left[H_{n}^{NEO}\right]}_{cc}=\partial^{2}V_{n}/\partial R_{c}^{2}$ , ${\left[H_{n}^{NEO}\right]}_{qc}=\partial^{2}V_{n}/\partial R_{n}\partial R_{c}$ , and ${\left[H_{n}^{NEO}\right]}_{qq}=\partial^{2}V_{n}/\partial R_{n}^{2}$ . This reduced Hessian matrix has been previously used in vibrational analysis^124^ and p*K*_a_ calculations.^68^

We calculate the | ***g***_*a*_ – ***g***_*b*_| term in eq 9 from the gradients obtained at the optimized MECP geometry. The reduced mass μ is calculated from the effective Hessian at the MECP^107^

21 $$H_{eff}=\frac{\left|g_{a}\right|H_{b}\pm \left|g_{b}\right|H_{a}}{\left|g_{a}\right|\pm \left|g_{b}\right|}$$

where the plus sign is used for peaked intersections and the minus sign is used for sloped intersections. The effective Hessian in eq 21 corresponds to the Hessian of the Lagrangian that has a minimum at the MECP, such that its eigenvalues are all positive (see the Supporting Information of ref 125 for the derivation). We then calculate the projected and mass-weighted Hessian^107,126^

22 $$H_{RC}=P_{RC}H_{eff}^{mw}P_{RC}$$

Here, **P**_RC_ projects onto the reaction coordinate and **H**_eff_^mw^ is the mass-weighted Hessian with elements ${\left[H_{eff}^{mw}\right]}_{ij}={\left[H_{eff}\right]}_{ij}/\sqrt{m_{i}m_{j}}$ , where *m*_*i*_ is the mass associated with coordinate *i*. The projection matrix is defined as

23 $$P_{RC}=uu^{T}$$

24 $$u=\frac{g_{a}^{mw}-g_{b}^{mw}}{\left|g_{a}^{mw}-g_{b}^{mw}\right|}$$

where the gradients are mass-weighted as ${\left[g_{n}^{mw}\right]}_{i}={\left[g_{n}\right]}_{i}/\sqrt{m_{i}}$ .

The projected Hessian has one nonzero eigenvalue associated with the eigenvector ***k*** ^mw^. We normalize ***k*** ^mw^ and transform it as $k_{i}=k_{i}^{mw}/\sqrt{m_{i}}$ . We can then calculate the reduced mass μ as^126^

25 $$\mu ={\left(k^{T}k\right)}^{-1}$$

In our current implementation, the reduced mass μ­(*R*), as well as the diabatic vibronic surface gradients ***g***_*a*_(*R*) and ***g***_*b*_(*R*), are assumed to be independent of the donor–acceptor distance *R*. These quantities are computed from the effective Hessian at the unconstrained optimized MECP geometry for each isotope. We tested the impact of this assumption by scaling the values of μ, ***g***_*a*_, and ***g***_*b*_ and found that the behavior of NEO-GRT is not sensitive to their specific values within a reasonable range (see Section S8). This analysis suggests that assuming these quantities to be independent of *R* does not introduce significant errors into our calculations. In future studies for higher temperature KIEs, where a broader range of *R* values may be accessible, this approximation could be relaxed. Lastly, we note that the effective Hessian given in eq 21 could also be used to calculate the vibrational partition function for the classical nuclei, after projecting out the translational, rotational, reaction coordinate, and donor–acceptor distance coordinate contributions.^126,127^

### 2.7 Workflow Summary

The general workflow for the KIE calculation is outlined in Figure 3, describing the types of calculations required, the quantities that are extracted from these calculations, and the related terms in the KIE expression. Although there are many different steps for this procedure, all of the calculations are straightforward and are computationally tractable for large molecular systems.

**Figure 3:**
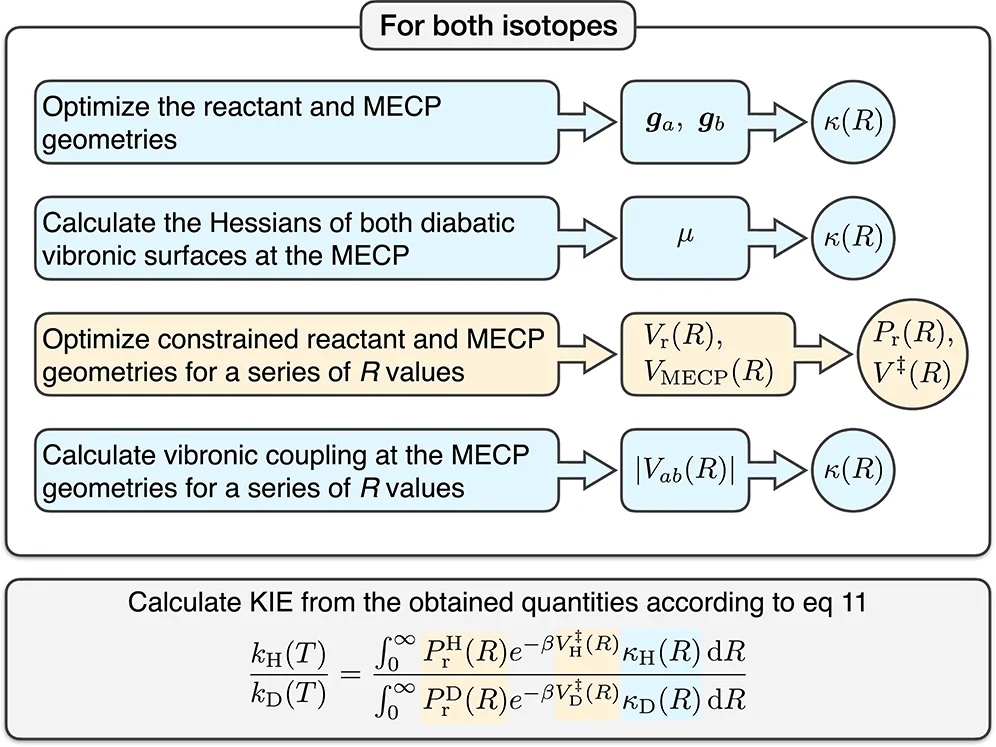
Overview of the KIE calculation workflow with NEO-GRT. For each isotope, NEO-DFT geometry optimizations and Hessian calculations, as well as NEO-MSDFT calculations of the vibronic couplings, are performed, and the input quantities are extracted from the results of these calculations. These quantities are then combined to compute the KIE. The temperature dependencies of *P*_r_ and κ are omitted for notational simplicity.

## 3 Computational Details

This section provides the computational details for the application of NEO-GRT, TST, and RPI theory to the two molecular systems studied. All quantum chemical calculations were performed with a developer version of Q-Chem 6.^128^

### 3.1 NEO-DFT and NEO-MSDFT Calculations

For the NEO-DFT calculations, we used the constrained NEO (CNEO) methodology,^129–131^ constraining the quantum proton position expectation value to the basis function center position. We chose CNEO-DFT because, in our experience, it tends to lead to faster geometry optimizations compared to regular NEO-DFT. All the calculations could be performed equivalently with NEO-DFT and are expected to give very similar results because at the complete basis set limit, the two approaches result in identical optimized geometries and energies due to mathematical equivalence. For the basis sets used herein, we have shown that the CNEO-DFT and NEO-DFT optimized geometries and energies are nearly identical (see Section S4). NEO-GRT is based on the NEO-DFT vibronic energy surface, which depends on only the classical nuclear coordinates, in contrast to the CNEO-DFT potential energy surface, which depends on the classical nuclear coordinates and the position expectation values of the quantum nuclei.^129^ In this work, we apply the constraint as a technical tool to facilitate convergence of the NEO-DFT geometry optimization, while still using the NEO-DFT vibronic energy surface. To avoid confusion about the type of vibronic energy surface used in NEO-GRT, we refer to the theory and results in this paper generally to be based on NEO-DFT.

We employed the ωB97X functional^132^ for electron exchange–correlation and the epc17-2 functional^64,65^ for electron–proton correlation. The ωB97X functional has been reported to accurately describe proton transfer reactions with conventional DFT.^133,134^ We note that for studying larger molecular systems, dispersion-corrected functionals may be warranted. For the theory-to-theory comparison with relatively small molecules performed herein, we expect London dispersion contributions to be insignificant. We used the def2-SVPD electronic basis set^135^ and the PB4-F2 protonic basis set.^136^ For calculations of the deuterium isotope, the protonic basis set exponents were scaled by $\sqrt{2}$ to account for the factor of 2 increase in the mass. The two-particle integral threshold was set to 10^–13^, and numerical integration used an unpruned (99, 590) Euler-Maclaurin-Lebedev quadrature. The simultaneous DIIS NEO-SCF solver was used;^137^ convergence was achieved when the energy change between consecutive iterations was below 10^–9^ *E*_h_ and the DIIS error below 10^–9^.

The NEO-MSDFT calculations also used CNEO-DFT with the same functionals and basis sets to obtain the localized diabatic vibronic states. We found that although both the electronic and protonic basis sets on the transferring hydrogen are considerably smaller than in previous quantitative studies,^69,70^ we could parametrize α and β (see eqs 13 and 14) to accurately reproduce numerically exact tunneling splittings calculated with the Fourier Grid Hamiltonian method^138,139^ for multiple small systems across a range of proton donor–acceptor distances (see Section S3). Separate α and β parameters were fitted for the hydrogen and deuterium isotopes. We note that the parameters for the H and D isotopes are relatively similar (Table S1). These parameters are also similar to previously obtained parameters for H with a different functional and basis set.^69^ A more comprehensive study is required to determine the level of transferability of these parameters across different basis sets and functionals. The results herein demonstrate that the NEO-MSDFT methodology can be used reliably with CNEO and for multiple isotopes.

We implemented the effective gradient shown in eq 17 in a developer version of Q-Chem and used it with the Q-Chem geometry optimization algorithm. The α_MECP_ value was set to 100 *E*_h_^–1^, and convergence was achieved when the maximum gradient component was below 10^–4^ *E*_h_/Bohr. These values typically led to an energy difference between the reactant and product diabatic vibronic states at the converged MECP below 10^–5^ *E*_h_ (0.006 kcal mol^–1^). In NEO-MSDFT calculations, the quantum proton is represented by two basis function centers optimized near the donor or acceptor, respectively. As a result, the basis sets used for the NEO-MSDFT calculations are slightly different from those used for the NEO-DFT calculations during MECP optimization, where the quantum proton is represented by only one basis function center. This difference in the basis sets leads to a slightly larger energy difference between the diabatic states in the NEO-MSDFT calculations, typically around 10^–4^ *E*_h_.

Processing the data, fitting analytical functions to computed *R*-dependent values, and other related calculations were performed with an openly available Python code (see Data and Software Availability Statement). The vibronic coupling values were fit using the following functional form

$$V_{ab}^{\prime }\left(R\right)=V_{0}\;\exp\left[c_{1}R+c_{2}R^{2}\right]$$ 26

where *V*_0_, *c*_1_, and *c*_2_ are fitting parameters. The quadratic term in the exponential has been found to be important for an accurate description of vibronic couplings for soft donor–acceptor modes and higher temperatures.^140^ The vibronic energy surfaces for the reactant and the MECP were fit to a fourth-order polynomial, subject to the constraint that the function remains convex for all values of *R*. The resulting parameters to the fitting functions are provided in Section S6. Different functional forms for *V*_r_(*R*) have been studied previously with one-dimensional models, showing some variation in the results.^96,97^ We found that the exact functional form used does not significantly impact the results as long as it enables a reasonable fit of the data.

Within the thermal averaging scheme, there exists a critical distance *R*_c_ below which the proton potential on the conventional electronic Born–Oppenheimer surface becomes a single well. For *R* < *R*_c_, the transferring proton is no longer in a double-well potential and thus cannot undergo transfer. To account for this situation, the transmission coefficient is set to zero [i.e., κ­(*T*, *R*) = 0] for *R* < *R*_c_, where *R*_c_ is defined as the distance at which the overlap *S*_*ab*_^′^(*R*) approaches unity. The detailed procedure for estimating *R*_c_ is provided in Section S5.

### 3.2 TST and RPI Calculations

We performed standard TST as well as ring-polymer instanton (RPI)^141–143^ theory calculations for comparison to the NEO-GRT KIE calculations. The conventional DFT calculations for TST and RPI utilized the same electronic functional and basis set as the NEO-DFT calculations (i.e., ωB97X and def2-SVPD). All optimizations and rate constant calculations for TST and RPI were performed with in-house Python codes provided by Jeremy Richardson (ETH Zurich), modified to interface with Q-Chem. Convergence was achieved when the maximum gradient component became less than 5 × 10^–5^ *E*_h_/Bohr. The number of beads for RPI was selected to be the lowest power of two that was above 40 × *T*_c_/*T*, where *T*_c_ is the crossover temperature estimated from the imaginary frequency at the transition state, and *T* is the temperature used for the simulation. An analogous approach has been shown to produce errors below 10%.^144^ For each reaction and isotope pair, RPI was optimized at temperatures as low as 50 K up to 20 K below the crossover temperature in 25 K steps. Additional computational details and the crossover temperatures are provided in Section S10.

## 4 Results and Discussion

We evaluated the NEO-GRT methodology by comparing the computed KIEs at various temperatures to those from conventional TST and RPI rate theory for the two intramolecular proton transfer reactions shown in Scheme 1. Although the KIEs for many proton transfer reactions have been measured experimentally, they typically occur in solvent and at room temperature, presumably involving contributions from vibrationally excited states. A direct comparison to experimental data requires further development of the theory and is the focus of ongoing work. Herein, we performed a theory-to-theory comparison to more cleanly evaluate the performance of NEO-GRT in the gas phase at relatively low temperatures. The RPI results are considered to be a highly accurate benchmark for these systems at the temperatures investigated. The two intramolecular proton transfer reactions studied herein are energetically favorable. A discussion of the reverse, energetically unfavorable reactions, together with an analysis of detailed balance, is provided in Section S9.

### 4.1 NEO-DFT Calculations

The computed vibronic surfaces and vibronic couplings as functions of the donor–acceptor distance *R* are shown for the two reactions given in Scheme 1 with both isotopes in Figure 4. The plots show that our selected functions fit the data well in the relevant regions. We note that at longer donor–acceptor distances (in these cases, above ∼2.75 Å), the vibronic coupling values from NEO-MSDFT become numerically noisy because the relevant integrals are computed between the overlapping tails of the protonic wave functions, where small numerical oscillations in the wave functions can cause relatively large changes in the computed values. Related issues have been observed in electron transfer studies with constrained DFT.^145^ However, those relatively large distances do not contribute significantly to the rate constants for these systems.

**Figure 4:**
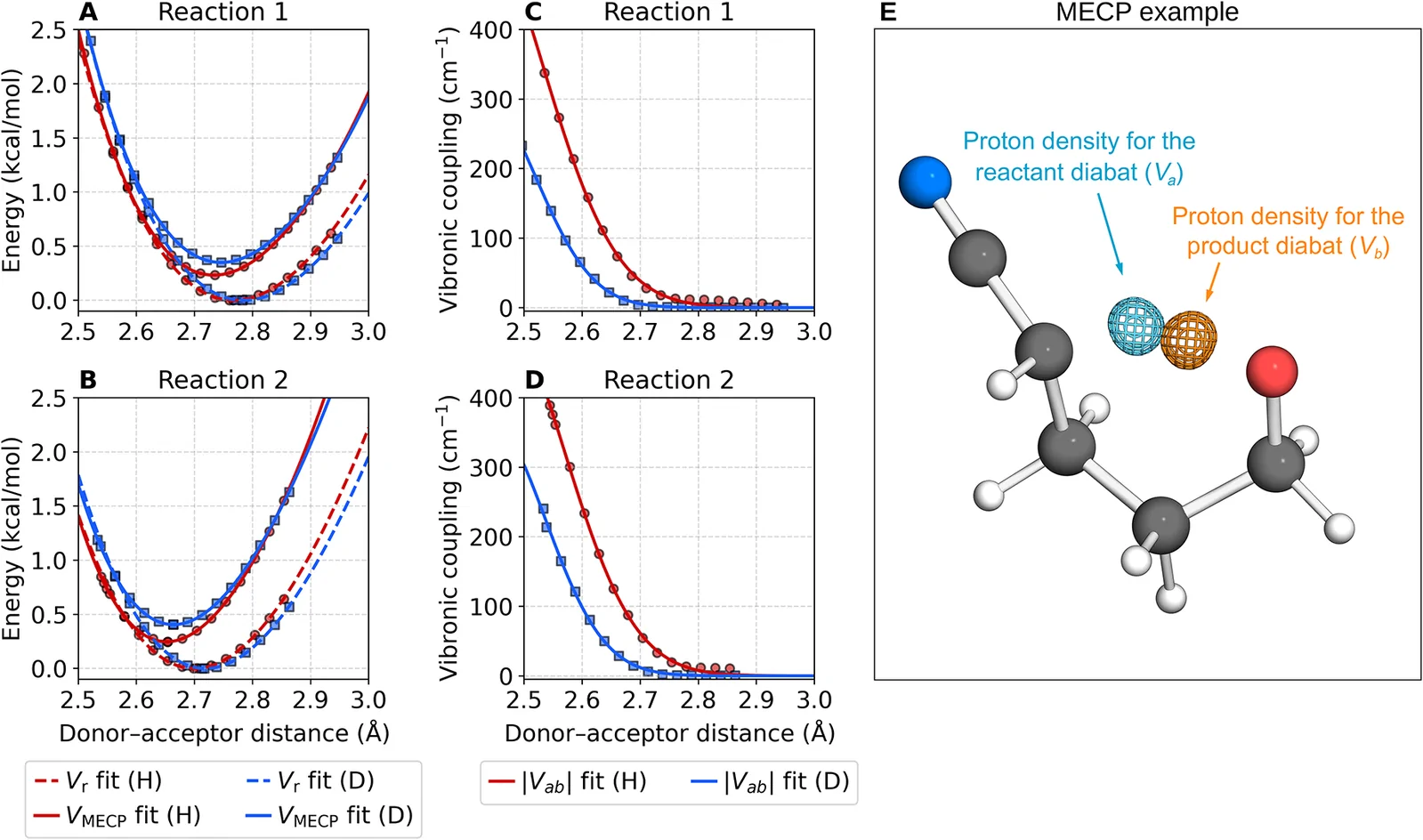
(A,B) Vibronic reactant and MECP energies as functions of the proton donor–acceptor distance *R*, calculated with NEO-DFT for Reaction 1 (A) and 2 (B). The zero of energy is set to the minimum of *V*_r_ for each case. (C,D) Vibronic couplings as functions of *R*, calculated with NEO-MSDFT for Reaction 1 (C) and 2 (D). (E) An example MECP geometry for Reaction 1 at *R* = 2.735 Å. The NEO-DFT proton densities of both diabatic states are shown with an isovalue of 0.01 au.

Both reactions exhibit a subtle geometric isotope effect, where the minimum energy donor–acceptor distance for the reactant is slightly shorter for H than for D by around 0.02 Å for Reaction 1 and 0.04 Å for Reaction 2 (compare minima of *V*_r_ curves for H and D in Figure 4A,B). As a result, the Boltzmann distributions are also slightly shifted, leading to different weighting of the donor–acceptor distances in the integrand of eq 11. As shorter distances generally correspond to higher rate constants due to the greater vibronic coupling values, this effect increases the H/D KIE. Another important factor is the ZPE, which is inherently included in NEO-DFT energies for the quantum nuclei. The vibronic activation energy is typically lower for H transfer than for D transfer due to a larger difference in the ZPE at the MECP than at the reactant for H compared to D (compare energies of *V*_MECP_ for H and D in Figure 4A and B). This ZPE effect also increases the H/D KIE. The NEO-DFT ZPEs can be corrected using single-point NEO coupled-cluster singles and doubles with perturbative triples (NEO-CCSD­(T))^146–148^ calculations, as discussed in Section S9. Such corrections do not significantly impact the KIEs for the forward reactions studied herein.

The exponential dependence of the vibronic coupling values on the donor–acceptor distance is shown in Figure 4C,D. Higher vibronic coupling values correspond to higher transition probabilities, as indicated by eq 9 for the LZ treatment or eq 10 for the WC treatment. Because the vibronic coupling values are higher for H than for D at all distances, the difference in vibronic coupling also results in increased H/D KIEs.

Figure 4E shows an example of an MECP geometry for Reaction 1 at *R* = 2.735 Å, along with the proton density for each diabatic vibronic state. Note that these proton densities were obtained from separate NEO-DFT calculations with the same classical nuclear coordinates but different quantum proton basis function center positions. The energy difference between these two localized NEO-DFT states is on the order of 10^–6^ *E*_h_ (approximately 6 × 10^–4^ kcal mol^–1^ or 0.2 cm^–1^), confirming that this geometry corresponds to a crossing point between the reactant and product diabatic vibronic surfaces.

### 4.2 KIE Comparison

The KIEs computed with the three methods used in this work are shown in Figure 5, and the numerical values are provided in Table S7. In these plots, the logarithm of the TST KIEs increases linearly as a function of inverse temperature, whereas the RPI KIEs, which include tunneling effects, show a nonlinear curve that plateaus at lower temperatures. For both reactions, the NEO-GRT KIE calculations agree well with the RPI calculated trends. Notably, the good agreement between the NEO-GRT and RPI calculations at low temperatures suggests that the NEO-GRT theory captures the impact of deep hydrogen tunneling on the KIEs. In addition, this agreement validates the underlying approximations of the NEO-GRT KIE calculations for these systems. These approximations include the treatment of only the transferring hydrogen nucleus fully quantum mechanically, the explicit treatment of the donor–acceptor coordinate under the assumption that it remains in thermal equilibrium, and the cancellation of the classical nuclear component of the H and D partition functions (see Section 2.3).

**Figure 5:**
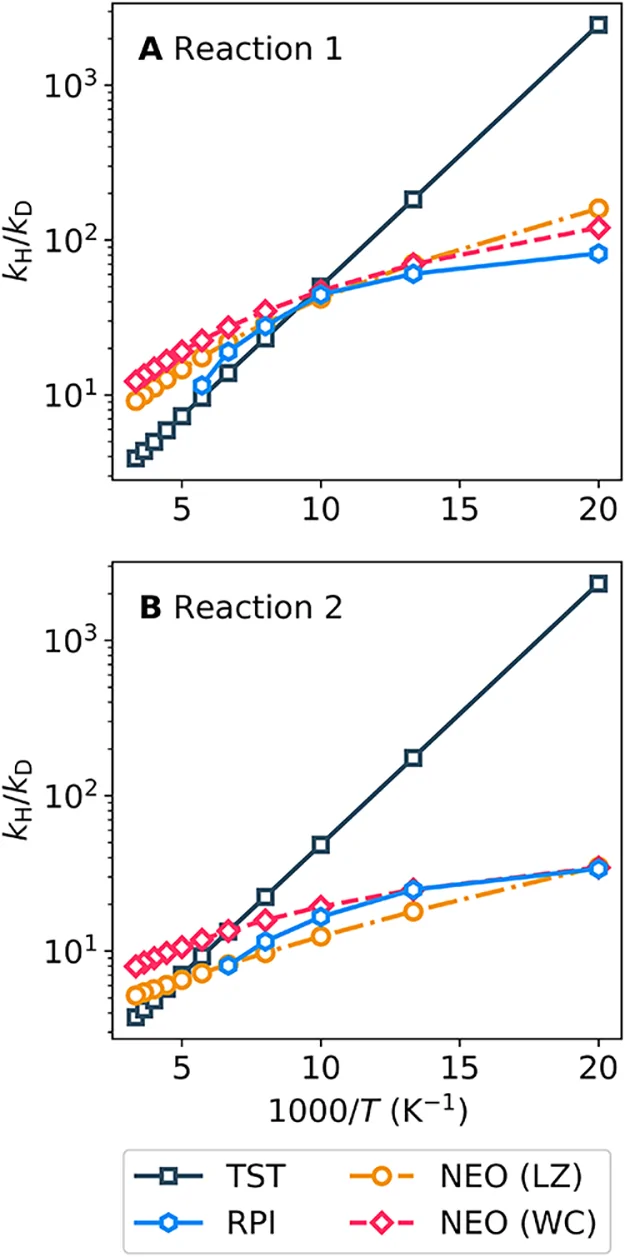
Logarithm of the computed H/D KIEs as a function of inverse temperature for Reaction 1 (A) and 2 (B) computed with NEO-GRT using the LZ or WC treatment, compared to TST and RPI results.

The agreement of the NEO-GRT results with the RPI results at lower temperatures is slightly better when the WC transmission coefficient rather than the LZ transmission coefficient is used. This difference suggests that the transferring isotope, namely H or D, also influences the tunneling of the other nuclei, which are not quantized with the NEO approach. In the WC formalism, which approximates the diabatic potential surfaces as linear functions,^108,112^ this isotope-dependent heavy-atom tunneling can be understood by considering the difference in the angle between the crossing surfaces for the H and D isotopes, which is captured by the |Δ ***g*** | term in eq 10. A smaller angle leads to a narrower barrier, accelerating the tunneling effect and leading to higher rate constants. The heavy-atom tunneling could be captured more accurately along the reaction coordinate using Zhu–Nakamura theory^108,149,150^ or in full dimensionality by combining NEO with nonadiabatic RPI theory.^151,152^ However, the good performance of the simpler LZ and WC approaches suggests that combining NEO-GRT with more sophisticated theories is probably not necessary for many applications.

In general, the choice between the WC and LZ transmission coefficients depends on the temperature range of interest and the physical characteristics of the system. We expect the WC approach to be more accurate in the low-temperature regime because it can capture some of the heavy-atom tunneling effects. In addition, the rate constant at lower temperatures is dominated by longer donor–acceptor distances, for which the vibronic coupling is smaller and the weak coupling approximation is likely to be valid. At higher temperatures, we expect the LZ approach to be more accurate because the heavy-atom tunneling effects are less important, and shorter donor–acceptor distances are thermally accessible. At these shorter distances, the vibronic coupling can be large enough such that the weak coupling approximation does not hold, in some cases even reaching the adiabatic limit, which can be captured by the LZ formalism. For similar reasons, the LZ transmission coefficient is likely to be more accurate for systems that sample shorter donor–acceptor distances due to either a shorter equilibrium distance or a more flexible hydrogen-bonding interface.

At higher temperatures, NEO-GRT deviates from RPI and TST. However, neither RPI nor TST are ideal benchmarks at higher temperatures: RPI is known to be less reliable close to the crossover temperature,^153,154^ and TST does not account for any tunneling effects. Nevertheless, it is also likely that NEO-GRT is inaccurate at higher temperatures, especially near room temperature. Model system calculations with related rate theories have shown that room-temperature KIE calculations may require inclusion of contributions from excited vibronic (i.e., proton vibrational) states.^91,98,101,103,104^ Current efforts are directed toward the inclusion of excited vibronic states within NEO-GRT. In addition, at higher temperatures, a wider range of donor–acceptor distances becomes accessible during the thermal averaging procedure, and the current assumption of constant ***g***_*n*_ and μ may become problematic, requiring additional calculations of these quantities as a function of *R*.

We emphasize that NEO-GRT is computationally efficient for computing KIEs of hydrogen transfer reactions. Calculation of the relevant input quantities requires NEO-DFT geometry optimizations for the reactant and MECP at a series of *R* values, two Hessian calculations at the MECP, and NEO-MSDFT calculations at each *R* value (see Figure 3). A NEO-DFT calculation is of comparable cost as the corresponding conventional DFT calculation, depending on the specific basis sets and algorithms, and a NEO-MSDFT energy calculation is a few times the cost of a single-point NEO-DFT energy calculation. For the KIE calculations reported here, we calculated data for up to 20 *R* values for each isotope, although we expect that similar quality results can be achieved with much fewer calculations. Moreover, once the relevant input quantities for NEO-GRT have been calculated, the KIE calculations at different temperatures require only computationally inexpensive postprocessing. In contrast, RPI theory requires separate instanton optimization and Hessian calculations for each temperature.

### 4.3 Future Directions

NEO-GRT can be extended in many different directions, especially for studying room temperature solution-phase hydrogen transfer reactions. As mentioned above, including excited vibronic states will be important for computing KIEs at room temperature. This extension will require a summation over pairs of reactant and product vibronic states, in conjunction with a NEO method for computing these excited vibronic states. In addition, solution-phase studies will require the inclusion of solvation effects. Although implicit solvent models have been implemented with NEO-DFT,^155–158^ they must also be implemented for NEO-MSDFT to calculate solution-phase vibronic couplings. To compute KIEs in enzymatic environments, a hybrid quantum mechanical/molecular mechanical method may be necessary.^157^ These directions are under current exploration in our group.

Although the NEO-MSDFT method produces accurate vibronic couplings over a wide range of donor–acceptor distances, it becomes less reliable at very long distances, corresponding to vibronic coupling values of less than around 5 cm^–1^. Typically these long distances and associated small vibronic couplings do not contribute significantly to the rate constant. For cases where such small vibronic couplings become relevant, we could use the NEO complete active space self-consistent field multireference configuration interaction (NEO-CASSCF/MRCI) approach,^159^ which is more expensive but has been shown to produce accurate hydrogen tunneling splittings for a wide range of systems and donor–acceptor distances.^114^

NEO-GRT could also be extended to compute absolute rate constants. In this case, the classical vibrational partition function and ZPE could be computed from the effective Hessian given in eq 21. However, the calculation of absolute rate constants is typically much more challenging than the calculation of KIEs.

## 5 Conclusions

In this work, we have introduced a nuclear–electronic orbital general rate theory (NEO-GRT) for calculating primary H/D KIEs. The current implementation focuses on electronically adiabatic hydrogen tunneling that can span the vibrationally adiabatic and nonadiabatic regimes. In this approach, the transferring hydrogen or deuterium nucleus is treated quantum mechanically on the same level as the electrons with NEO-DFT to produce diabatic vibronic states that depend on the coordinates of the other nuclei. These diabatic vibronic states are used as the basis for a general rate constant theory that spans the adiabatic and nonadiabatic limits and the intermediate regime. The tunneling probability of the transferring hydrogen or deuterium nucleus is described via the square of the vibronic coupling, which is computed with NEO multistate DFT at the minimum energy crossing point. The tunneling of the other nuclei along the reaction coordinate can also be included in the weak coupling limit. A thermal averaging procedure is used to account for the fluctuations of the proton donor–acceptor distance, which plays an important role in hydrogen transfer reactions because the vibronic coupling depends strongly on this distance. A practical prescription for computing all of the input quantities for the resulting rate constant expression is provided.

We evaluated the performance of NEO-GRT for two intramolecular proton transfer reactions by comparison to RPI theory, which is expected to be accurate for these systems at low temperatures. Both types of calculations were performed for the full-dimensional molecular systems at the same level of electronic structure theory. The agreement between the KIEs computed with NEO-GRT and RPI theory for the low-temperature range down to 50 K provides validation for NEO-GRT. Specifically, this agreement shows that NEO-GRT captures the deep hydrogen tunneling effects necessary to compute these KIEs accurately. Future work will focus on extending the applicability domain of NEO-GRT to solution-phase reactions at room temperature.

## Supplementary Material



## Data Availability

The data underlying this study are openly available on Zenodo at 10.5281/zenodo.21603561. The code for NEO-GRT calculations is available in the Zenodo repository and on Github at https://github.com/shsgroup/NEO-GRT.

## Notes

Derivation of the rate constant expression; description of the design of Reactions 1 and 2; NEO-MSDFT scaling scheme and parametrization; comparison of NEO-DFT and CNEO-DFT optimized geometries; critical distance estimation; parameters for fitting functions; numerical results for the data in Figure 5; sensitivity analysis; detailed balance and reverse reaction analysis; additional computational details for RPI (PDF)
