# Supplementary material for: Nuclear–Electronic Orbital General Rate Theory: Predicting Hydrogen Kinetic Isotope Effects in the Deep Tunneling Regime
Source: J Chem Theory Comput. 2026 Aug 21;22(17):8836–50. doi: 10.1021/acs.jctc.6c01175 (PMC13564363; doi:10.1021/acs.jctc.6c01175)
Supplement: Supplementary file 1 [file ct6c01175_si_001.pdf]

Supporting Information

Nuclear–Electronic Orbital General Rate Theory: Predicting Hydrogen Kinetic Isotope Effects in the Deep Tunneling Regime

Eno Paenurk, Jang Mok Yoo, and Sharon Hammes-Schiffer*

Department of Chemistry, Princeton University, Princeton, NJ 08544, United States

E-mail: shs556@princeton.edu

Contents

S1 Derivation of the Rate Expression **S3**

S2 Design of Molecular Reactions **S5**

S3 NEO-MSDFT **S5**

S3.1 Theoretical Background S5

S3.2 NEO-MSDFT Scaling Scheme S9

S3.3 NEO-MSDFT Parameterization of Scaling Scheme S10

S3.3.1 Parameterization Systems S10

S3.3.2 Computational Details S11

S3.3.3 Fitting S12

S3.3.4 Analysis Justifying New Scaling Scheme S14

S4 Comparison of NEO-DFT and CNEO-DFT Optimized Geometries **S18**

S5 Critical Distance Estimation **S18**

S6 Parameters for Fitting Functions **S19**

S7 Numerical Results **S20**

S8 Sensitivity Analysis **S21**

S9 Detailed Balance and Reverse Reaction Analysis **S22**

S9.1 Detailed Balance S22

S9.2 Results for Reverse Reactions S24

S10 RPI Additional Computational Details **S30**

References **S32**

S1 Derivation of the Rate Expression

Starting from the canonical rate constant expression for nonadiabatic statistical theory,[1,2] the rate constant at a fixed donor–acceptor distance R can be written as

$$k(T,R) = \frac{1}{h}\frac{Q^{\ddagger}(T,R)}{Q_r(T,R)} \times \int_0^{\infty} P_{\mathrm{trans}}(\varepsilon,R)\, e^{-\beta\varepsilon}\, \mathrm{d}\varepsilon \tag{S1}$$

where $V_{\mathrm{r}}(R) = 0$. Substituting $\varepsilon = V^{\ddagger}(R) + E$, where $V^{\ddagger}(R) = V_{\mathrm{MECP}}(R) - V_{\mathrm{r}}(R)$ (see Figure S1), yields

$$k(T,R) = \frac{1}{\beta h}\frac{Q^{\ddagger}(T,R)}{Q_r(T,R)} \times e^{-\beta V^{\ddagger}(R)} \times \underbrace{\beta \int_{-V^{\ddagger}(R)}^{\infty} P_{\mathrm{trans}}(E,R)\, e^{-\beta E}\, \mathrm{d}E}_{\kappa(T,R)} \tag{S2}$$

Here, we have multiplied the expression by $\beta^{-1}\beta = 1$, which allows for a definition of $\kappa(T,R)$ that equals unity in the classical adiabatic limit. We can verify this limit by defining $P_{\mathrm{trans}}(E,R)$ as the Heaviside step function $\Theta(E)$, where the transmission probability is 0 for $E < 0$ and 1 for $E > 0$. (Note that $E < 0$ corresponds to $\varepsilon < V^{\ddagger}(R)$, i.e., the energy associated with the reaction coordinate is less than the barrier height.) In this case,

$$\kappa^{\mathrm{adiabatic}}(T,R) = \beta \int_{-V^{\ddagger}(R)}^{\infty} \Theta(E)\, e^{-\beta E}\, \mathrm{d}E = \beta\frac{1}{\beta} = 1 \tag{S3}$$

When $\kappa(T,R) = 1$, the remaining terms in eq S2 give the standard TST rate constant expression.

To arrive at the Landau–Zener (LZ) transmission coefficient $\kappa^{\mathrm{LZ}}(T,R)$, we first note that the LZ transition probability (eq 9 in the main text) is formulated for classical trajectories that reach the crossing region with $E > 0$, i.e., it does not account for tunneling of the non-quantized nuclei. To transform the energy domain integral into a velocity domain integral, we can substitute $E = \frac{1}{2}\mu(R)v^2$ such that $\mathrm{d}E = \mu v\, \mathrm{d}v$, where μ is the reduced mass associated with the reaction coordinate. We then note that $v(E) = \sqrt{2E/\mu}$ and thus define $P_{\mathrm{trans}}(E) =$

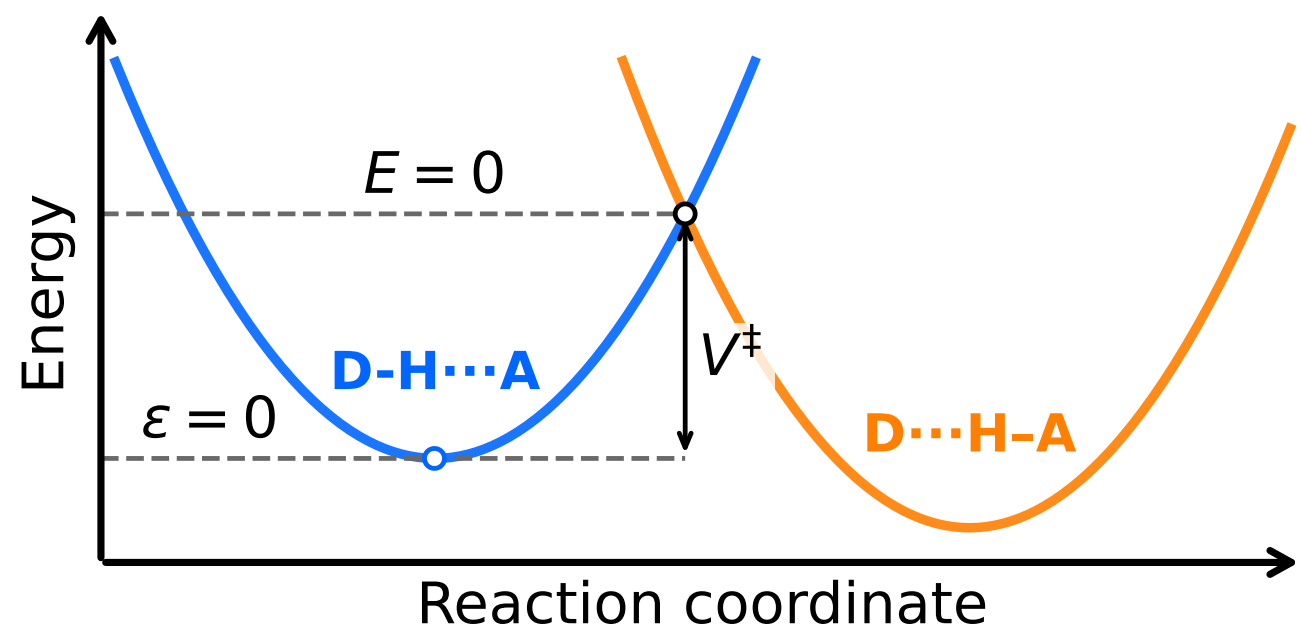

Figure S1. Energy level definitions shown for a schematic illustration of the vibronic surfaces. See Figure 1 in the main text for a detailed description of the surfaces.

$P_{\text{trans}}(v(E)) \equiv P^{\text{LZ}}_{\text{trans}}(v)$. Here we are assuming the LZ transition probability given by eq 9 in the main text. Together, these relations lead to

$$\kappa^{\text{LZ}}(T,R) = \beta \int_{-V^{\ddagger}(R)}^{\infty} P_{\text{trans}}(E,R)\, e^{-\beta E}\, \mathrm{d}E \tag{S4a}$$

$$= \beta \left[\underbrace{\int_{-V^{\ddagger}(R)}^{0} P_{\text{trans}}(E,R)\, e^{-\beta E}\, \mathrm{d}E}_{0} + \int_{0}^{\infty} P_{\text{trans}}(E,R)\, e^{-\beta E}\, \mathrm{d}E \right] \tag{S4b}$$

$$= \beta \int_{0}^{\infty} P_{\text{trans}}(E,R)\, e^{-\beta E}\, \mathrm{d}E \tag{S4c}$$

$$= \beta\mu \int_{0}^{\infty} v P^{\text{LZ}}_{\text{trans}}(v,R)\, e^{-\frac{1}{2}\beta\mu v^2}\, \mathrm{d}v \tag{S4d}$$

which is the velocity-averaged transmission coefficient based on Kuki's distribution as given in eq 7 in the main text. Using the Landau–Zener transmission coefficient reduces the rate constant to the TST expression in the large coupling limit and to a Golden Rule type expression in the weak coupling limit.[3]

We note that by calculating the canonical rate constants, we implicitly assume that the system is coupled to a thermal bath. For solution-phase reactions at room temperature, the thermal bath assumption is generally valid, and the canonical rate constant is well-defined. For the gas-phase reactions studied herein, the molecule could be implicitly assumed to be weakly coupled to a thermal bath. Although this assumption might not hold for

experimentally studied low-temperature gas-phase reactions, we can make this assumption for a purely theoretical comparison of thermal rate theories.

S2 Design of Molecular Reactions

We studied the intramolecular proton transfer reaction of two anions: 4-cyanobutanolate and 4,4-dicyanobutanoate, corresponding to Reaction 1 and Reaction 2, respectively, in Scheme 1 in the main text. The butane backbone was inspired by experimental studies in which KIEs were measured for intramolecular deprotonations of nitroalkanes with a butane framework[4] or an aromatic framework with four carbons between the donor and acceptor groups.[5] Although nitroalkane deprotonations are some of the most extensively studied examples of large KIEs and hydrogen tunneling,[6–11] cyanoalkane deprotonation reactions have been found to exhibit unusually large KIEs as well.[12] To keep the molecular structures as simple as possible, we chose to utilize cyano substituents instead of nitro substituents in our reaction design. The specific donor and acceptor groups were designed such that C-H bond cleavage is energetically favorable, while both the reactant and product structures correspond to minima on the vibronic energy surface. For this reason, the stronger C-H acid with the dicyano substituent was paired with a weakly basic carboxylate group, and the weaker C-H acid with one cyano substituent was paired with a strongly basic olate group. The reaction energies on the NEO-DFT vibronic surface with the hydrogen isotope are -1.0 kcal mol^{-1} for Reaction 1 and -2.3 kcal mol^{-1} for Reaction 2.

S3 NEO-MSDFT

S3.1 Theoretical Background

We start with an overview of the main equations of nuclear–electronic orbital multistate density functional theory (NEO-MSDFT) for the two-state problem, as presented in ref

13. Here, we consider a hydrogen transfer system in a symmetric or nearly symmetric environment. In this case, two localized nuclear–electronic Kohn–Sham wavefunctions, $|\Psi_\text{I}\rangle$ and $|\Psi_\text{II}\rangle$, are obtained from NEO-DFT. Each localized wavefunction is the product of an electronic and a protonic determinant: $|\Psi_\text{i}\rangle = \Phi_i^\text{e}\Phi_i^\text{p}$. Using a nonorthogonal configuration interaction approach, the ground and first excited state vibronic wavefunctions are linear combinations of these localized wavefunctions:

$$|\Psi_0\rangle = D_\text{I}^0\,|\Psi_\text{I}\rangle + D_\text{II}^0\,|\Psi_\text{II}\rangle \tag{S5a}$$

$$|\Psi_1\rangle = D_\text{I}^1\,|\Psi_\text{I}\rangle + D_\text{II}^1\,|\Psi_\text{II}\rangle \tag{S5b}$$

The coefficients are determined by solving the 2×2 NEO-MSDFT matrix equation:

$$\mathbf{HD} = \mathbf{SDE} \tag{S6}$$

where

$$\mathbf{H} = \begin{bmatrix} H_\text{I,I} & H_\text{I,II} \\ H_\text{II,I} & H_\text{II,II} \end{bmatrix} \tag{S7}$$

and

$$\mathbf{S} = \begin{bmatrix} S_\text{I,I} & S_\text{I,II} \\ S_\text{II,I} & S_\text{II,II} \end{bmatrix} = \begin{bmatrix} 1 & \langle\Psi_\text{I}|\Psi_\text{II}\rangle \\ \langle\Psi_\text{II}|\Psi_\text{I}\rangle & 1 \end{bmatrix} \tag{S8}$$

The off-diagonal elements of the overlap matrix $\mathbf{S}$ are

$$S_\text{I,II} = \langle\Psi_\text{I}|\Psi_\text{II}\rangle = \langle\Phi_\text{I}^\text{e}\Phi_\text{I}^\text{p}|\Phi_\text{II}^\text{e}\Phi_\text{II}^\text{p}\rangle = S_\text{I,II}^\text{e}S_\text{I,II}^\text{p} \tag{S9}$$

where $S_\text{I,II}^\text{e}$ and $S_\text{I,II}^\text{p}$ are the overlap integrals of the electronic and protonic wavefunctions, respectively.

The diagonal elements of $\mathbf{H}$ are the NEO-DFT energies of the localized solutions. The

off-diagonal elements can be calculated as[13]

$$H_{\text{I,II}} = \langle \Psi_{\text{I}} | \hat{H}^{\text{NEO}} | \Psi_{\text{II}} \rangle + \frac{1}{2} S_{\text{I,II}} (E_{\text{I}}^{\text{corr}} + E_{\text{II}}^{\text{corr}}) \tag{S10}$$

where the first term is the energy computed at the NEO-HF level with the NEO-DFT determinants, and $E_{\text{I}}^{\text{corr}}$ and $E_{\text{II}}^{\text{corr}}$ are the correlation energies calculated as the difference between the NEO-DFT and NEO-HF energies calculated with the respective NEO-DFT determinants. Note that the first term can be expressed as a product of the overlap and energy terms associated with the transition density matrix (see eq 8 in ref 13), such that

$$\langle \Psi_{\text{I}} | \hat{H}^{\text{NEO}} | \Psi_{\text{II}} \rangle = S_{\text{I,II}} \varepsilon_{\text{I,II}}^{\text{NEO}} \tag{S11}$$

where $\varepsilon_{\text{I,II}}^{\text{NEO}}$ represents the sum of NEO-HF energy terms associated with the transition density matrix.

The vibronic coupling between the symmetrically orthogonalized localized NEO-DFT states is given by[13]

$$V = \frac{1}{1 - S_{\text{I,II}}^2} \left| H_{\text{I,II}} - \frac{(H_{\text{I,I}} + H_{\text{II,II}})}{2} S_{\text{I,II}} \right| \tag{S12}$$

To simplify this equation, we express $H_{\text{I,II}}$ as a product of $S_{\text{I,II}}$ and an energy term:

$$\begin{aligned} H_{\text{I,II}} &= \langle \Psi_{\text{I}} | \, \hat{H}^{\text{NEO}} \, | \Psi_{\text{II}} \rangle + \frac{1}{2} S_{\text{I,II}} (E_{\text{I}}^{\text{corr}} + E_{\text{II}}^{\text{corr}}) \\ &= S_{\text{I,II}} \varepsilon_{\text{I,II}}^{\text{NEO}} + \frac{1}{2} S_{\text{I,II}} (E_{\text{I}}^{\text{corr}} + E_{\text{II}}^{\text{corr}}) \\ &= S_{\text{I,II}} E'_{\text{I,II}} \end{aligned} \tag{S13}$$

where $E'_{\mathrm{I,II}} = \varepsilon_{\mathrm{I,II}}^{\mathrm{NEO}} + 0.5(E_{\mathrm{I}}^{\mathrm{corr}} + E_{\mathrm{II}}^{\mathrm{corr}})$. We can then write eq S12 as

$$\begin{aligned} V &= \frac{1}{1 - S_{\mathrm{I,II}}^2} \left| S_{\mathrm{I,II}} E'_{\mathrm{I,II}} - \frac{(H_{\mathrm{I}} + H_{\mathrm{II}})}{2} S_{\mathrm{I,II}} \right| \\ &= \frac{S_{\mathrm{I,II}}}{1 - S_{\mathrm{I,II}}^2} \left| E'_{\mathrm{I,II}} - \frac{(H_{\mathrm{I}} + H_{\mathrm{II}})}{2} \right| \\ &= \frac{S_{\mathrm{I,II}}}{1 - S_{\mathrm{I,II}}^2} |V'| \end{aligned} \tag{S14}$$

where $|V'| = |E'_{\mathrm{I,II}} - 0.5(H_{\mathrm{I}} + H_{\mathrm{II}})|$.

Previous work found that NEO-MSDFT did not reproduce the tunneling splittings calculated with the Fourier Grid Hamiltonian (FGH) method, which is numerically exact for electronically adiabatic systems. This deviation was hypothesized to stem from overly localized protonic wavefunctions, presumably due to limitations of the epc17-2 electron–proton correlation functional,[14,15] resulting in too small overlap values. As a solution, a corrected overlap form was suggested,

$$S_{\mathrm{I,II}}^{\mathrm{corr}} = \alpha (S_{\mathrm{I,II}})^{\beta} \tag{S15}$$

The parameters were fitted to reproduce FGH results for FHF^- at various F–F distances, leading to $\alpha = 0.0604$ and $\beta = 0.492$ for B3LYP[16–18]/aug-cc-pVDZ[19](aug-cc-pV5Z[20] for quantum proton) and epc17-2[14,15]/PB5-G.[21] NEO-MSDFT in conjunction with these parameters was found to reproduce the FGH tunneling splittings for multiple molecules at varying geometries,[13] even extending to systems with multiple quantum protons.[22]

Although this approach worked well for moderate to long donor–acceptor distances, we found that this original parameterization scheme was not quantitatively accurate for shorter donor–acceptor distances. The NEO-GRT theory presented herein requires integration over a wide range of donor–acceptor distances, thereby requiring the vibronic couplings at shorter distances to be quantitatively accurate. To address this issue, we devised an alternative scaling scheme that also uses only two parameters but is more broadly applicable to shorter donor–acceptor distances.

S3.2 NEO-MSDFT Scaling Scheme

In this section, we present the new NEO-MSDFT scaling scheme. Our analysis of the distance dependence of the vibronic coupling computed with the numerically exact FGH method for several model systems confirms that the overlap should be modified by the power β, as in the original parameterization scheme (eq S15). However, this analysis indicates that the α that was a multiplicative constant to the overlap in the original scheme (eq S15) should multiply the $|V'|$ term instead. In other words, instead of the vibronic coupling expression with the original overlap correction (eq S15)

$$V_{\text{orig}}^{\text{corr}} = \frac{\alpha S_{\text{I,II}}^{\beta}}{1 - \alpha^2 S_{\text{I,II}}^{2\beta}} |V'| \tag{S16}$$

our analysis leads to the following scaled vibronic coupling expression:

$$V_{\text{new}}^{\text{corr}} = \frac{S_{\text{I,II}}^{\beta}}{1 - S_{\text{I,II}}^{2\beta}} \alpha |V'| \tag{S17}$$

These expressions have the same $S_{\text{I,II}} \to 0$ limit. Thus, in the small overlap regime (i.e., long distance regime), the two corrections are expected to give similar results. A comparison of the two schemes for physically reasonable values of the parameters is shown in Figure S2. They agree quite well for distances in the range $r > 0.4$ Å. At closer distances, however, the functions differ significantly. The new scaling scheme is more physically realistic at shorter distance, where it plateaus rather than decreases as r decreases. We emphasize that this analysis with approximate functions is only illustrative and is presented to motivate eq S17 as the new scaling scheme.

The α and β values were parameterized by fitting the tunneling splittings calculated with NEO-MSDFT to those calculated with the FGH method. We tested the parameters with both the original (eq S16) and the new (eq S17) scaling schemes. Further physical and mathematical justification for the new scaling scheme is given below in Section S3.3.4.

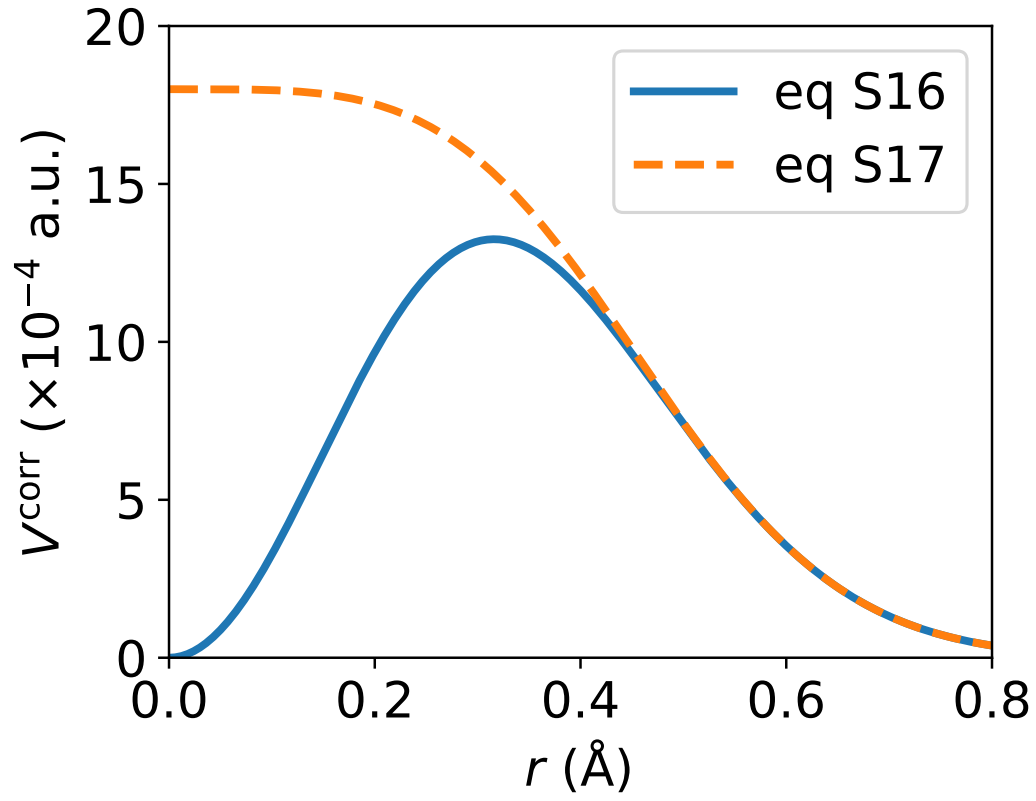

Figure S2. Comparison of V^{corr} from eqs S16 and S17 with $S = e^{-ar^2}$ and $a = 20$, $|V'| = br^2$ and $b = 0.6$, and $\alpha = 0.06$ and $\beta = 0.5$ as representative parameters.

S3.3 NEO-MSDFT Parameterization of Scaling Scheme

S3.3.1 Parameterization Systems

Our goal is to parameterize NEO-MSDFT with a few small model systems by fitting the tunneling splittings to numerically exact FGH results. The α and β parameters will then be fixed for all future calculations, analogous to a DFT functional. Given the transferability observed for NEO-MSDFT with the original scaling scheme,[13,22] we expect these parameters to be broadly transferable with the new scaling scheme.

We chose three symmetric proton transfer systems for this parameterization. These systems were selected to cover the three most common proton donor and acceptor atoms (C, N, and O), such that the resulting parameters could be expected to work well with various organic proton transfer reactions. In addition, these systems are electronically adiabatic, thereby enabling direct comparison to the FGH results, which are numerically exact for electronically adiabatic systems.

For each dimer in Figure S3, calculations were performed at multiple heavy-atom distances. These donor–acceptor distances were selected such that the highest tunneling splitting with the hydrogen isotope was below 600 cm^{-1} and the lowest tunneling splitting with the deuterium isotope was above 1 cm^{-1}. The selected distances were (in Å) $[CH_4CH_3]^-$:

2.80, 2.85, 2.90, 2.95, 3.00; $[NH_4NH_3]^+$: 2.70, 2.75, 2.80, 2.85, 2.90; $[H_3OH_2O]^+$: 2.60, 2.65, 2.70, 2.75, 2.80.

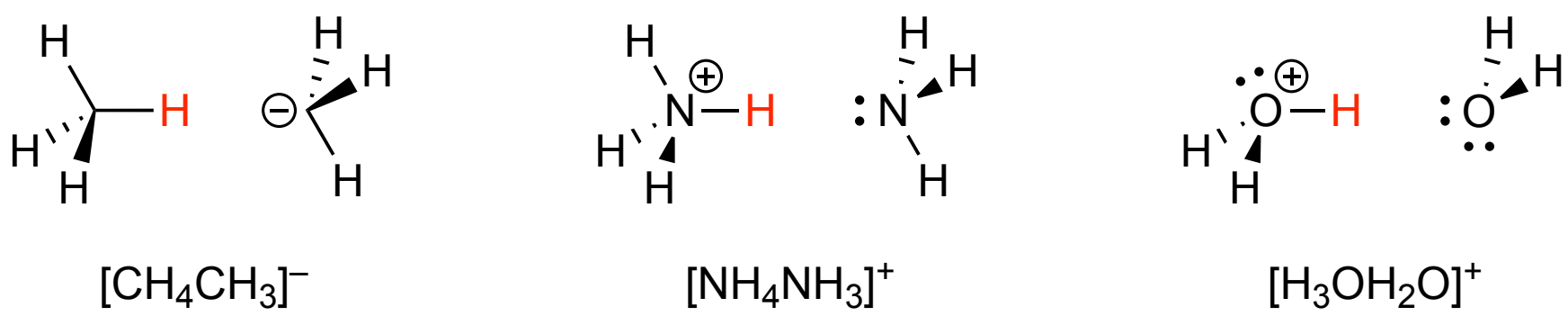

Figure S3. Proton transfer dimers used for parameterization. The quantized proton is shown in red.

S3.3.2 Computational Details

The dimer geometries were prepared by first optimizing the protonated monomer geometry for each dimer, i.e., CH_4, NH_4^+, and H_3O^+. The optimizations were performed with the ωB97X functional[23] and aug-cc-pVDZ basis set.[19] The default geometry optimization thresholds were used. The self-consistent field (SCF) procedure was solved with the GDM algorithm, the convergence threshold was set to RMS gradient below 10^{-8}, the two-particle integral threshold was set to 10^{-12}, and unpruned (75, 302) Euler-Maclaurin-Lebedev quadrature for numerical integration was used. The optimized monomer geometries were symmetrized with the `point_charge_symmetry` code.[24] For each dimer and distance, the two monomer geometries were combined such that the geometries of the non-quantized atoms belonged to the point groups D_{3d} for $[CH_4CH_3]^-$ and $[NH_4NH_3]^+$ and C_{2h} for $[H_3OH_2O]^+$.

The reference tunneling splitting values were calculated with the Fourier Grid Hamiltonian (FGH) method,[25] using an in-house code of the FGH multiconfigurational SCF (MC-SCF) method.[26] The potential energy grid was generated by moving the transferring proton on a three-dimensional grid while keeping the rest of the dimer geometry fixed. The single-point DFT energy was calculated at each point with the ωB97X functional and def2-SVPD basis set,[27] using the GDM SCF solver, SCF convergence threshold of 10^{-10}, two-particle integral threshold of 10^{-14}, and unpruned (99, 590) quadrature. The three-dimensional grid spanned from -0.93 to 0.93 Å with 32 grid points in each direction, corresponding to 0.06 Å

spacing between the points and 32,768 points in total. In practice, only 8192 unique points were calculated, and the full grid was constructed considering the symmetry of the dimers. The two lowest states were calculated with FGH-MCSCF, including 25 states per dimension and using mass 1836.1527 a.u. for the H isotope and 3670.4830 a.u. for the D isotope.[28] The tunneling splitting was calculated as the energy difference between these states.

For the NEO-MSDFT calculations, the quantum proton basis function center position was first optimized with the symmetric dimer geometry, keeping the coordinates of the non-quantized atoms fixed. We used CNEO-DFT with the ωB97X electronic functional and the def2-SVPD electronic basis set, as well as the epc17-2 electron–proton correlation functional[14,15] and the PB4-F2 protonic basis set.[21] For calculations of the deuterium isotope, the protonic basis set exponents were scaled by $\sqrt{2}$. The two-particle integral threshold was set to 10^{-14}, and numerical integration used an unpruned (99, 590) quadrature. The simultaneous DIIS NEO-SCF solver was used[29] with convergence thresholds of energy change between consecutive iterations below 10^{-10} Hartree and the DIIS error below 10^{-10}. The geometry optimization converged when the largest gradient component was below 10^{-4} a.u. The optimized quantum proton basis function center position was then mirrored such that in the NEO-MSDFT calculation, there were two symmetrically placed basis function centers for the quantum proton. The diabatic CNEO-DFT states were calculated in this expanded basis set, with the position expectation value constrained to either of the basis function center positions. The NEO-MSDFT calculation was performed with the same SCF settings as used in the CNEO-DFT optimization, and the Hamiltonian and overlap matrix elements were extracted (eqs S7 and S8).

S3.3.3 Fitting

The α and β values were parameterized by fitting the tunneling splittings calculated with NEO-MSDFT to those from FGH. We performed this fitting procedure with both the original and the new scaling schemes. A comparison between the reference FGH data and the result-

ing NEO-MSDFT tunneling splittings is shown in Figure S4, and the resulting parameters are given in Table S1.

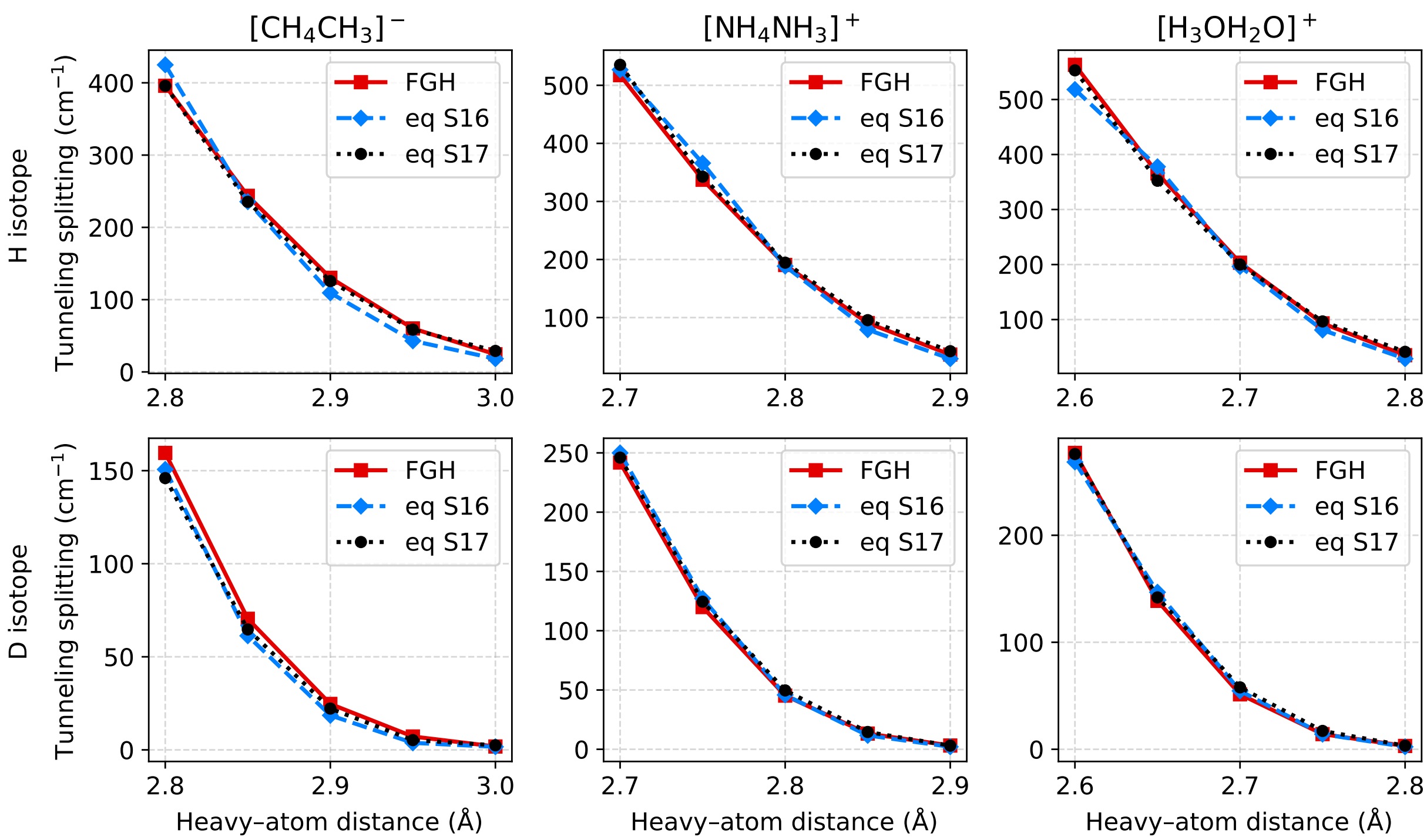

Figure S4. FGH reference and NEO-MSDFT tunneling splittings using the scaling schemes given by eqs S16 and S17.

Table S1. Fitted Parameters for NEO-MSDFT Tunneling Splittings and RMSD with Respect to FGH Reference Values[a]

Scaling scheme	Isotope	α	β	RMSD / cm^{-1}
eq S16	H	0.090	0.612	18.5
eq S16	D	0.057	0.595	5.7
eq S17	H	0.062	0.522	7.6
eq S17	D	0.045	0.544	4.8

[a] The NEO-MSDFT calculations were performed with ωB97X/def2-SVPD and epc17-2/PB4-F2, and the reference FGH calculations were performed with ωB97X/def2-SVPD.

This comparison shows that the new coupling correction equation (eq S17) enables better fits of the reference FGH data than the original scaling scheme (eq S16). The improvement seen here is more significant for the H isotope, presumably because the overlap values are

larger than those for the D isotope, such that they are in the regime where the two schemes differ (cf. Figure S2). We expect similar improvement for any isotope at short enough distances. Because the KIE calculation requires integrating over the donor–acceptor distances and short distances can have a dominant impact on the calculated rate constants, it is important to have accurate vibronic coupling values at these short distances. Thus, in this work, we used eq S17 with the respective parameters listed in Table S1.

S3.3.4 Analysis Justifying New Scaling Scheme

In this subsection, we analyze the behavior of the NEO-MSDFT and FGH results for the model systems described above to justify the new scaling scheme. According to eq S14, the vibronic coupling depends on the overlap $S_{\mathrm{I,II}}$ and the energy term $|V'|$. Figures S5 and S6 show how these quantities change as a function of the distance between the proton position expectation values of the localized diabatic states. This distance can be expressed as $\delta \langle r \rangle = |\, \langle r \rangle_{\mathrm{I}} - \langle r \rangle_{\mathrm{II}} \,|$. Both the natural logarithm of $S_{\mathrm{I,II}}$ and $|V'|$ can be fit well with $y = c_2 x^2 + c_0$. The plots for the D isotope show that the NEO-MSDFT calculations become numerically noisy at longer distances, as indicated by the overlap in Figure S5 and $|V'|$ in Figure S6.

We can use the quadratic functional forms of $\ln(S_{\mathrm{I,II}})$ and $|V'|$ to construct a simple expression for how the vibronic coupling changes with the distance between the proton position expectation values of the localized wavefunctions. To simplify the notation, we take $r \equiv \delta \langle r \rangle$. Regarding the overlap, the negative c_2 coefficients in Figure S5 indicate that $\ln(S_{\mathrm{I,II}}) \propto ar^2$, and, therefore, $S_{\mathrm{I,II}} \propto e^{-ar^2}$, where a is a positive constant. This relation would be exact if Ψ_{I} and Ψ_{II} were Gaussian functions. The magnitude of a quantifies the widths of Ψ_{I} and Ψ_{II} – a larger value of a corresponds to more localized functions for which the overlap decays faster with increasing distance. Considering that $|V'| \propto br^2$, we write an

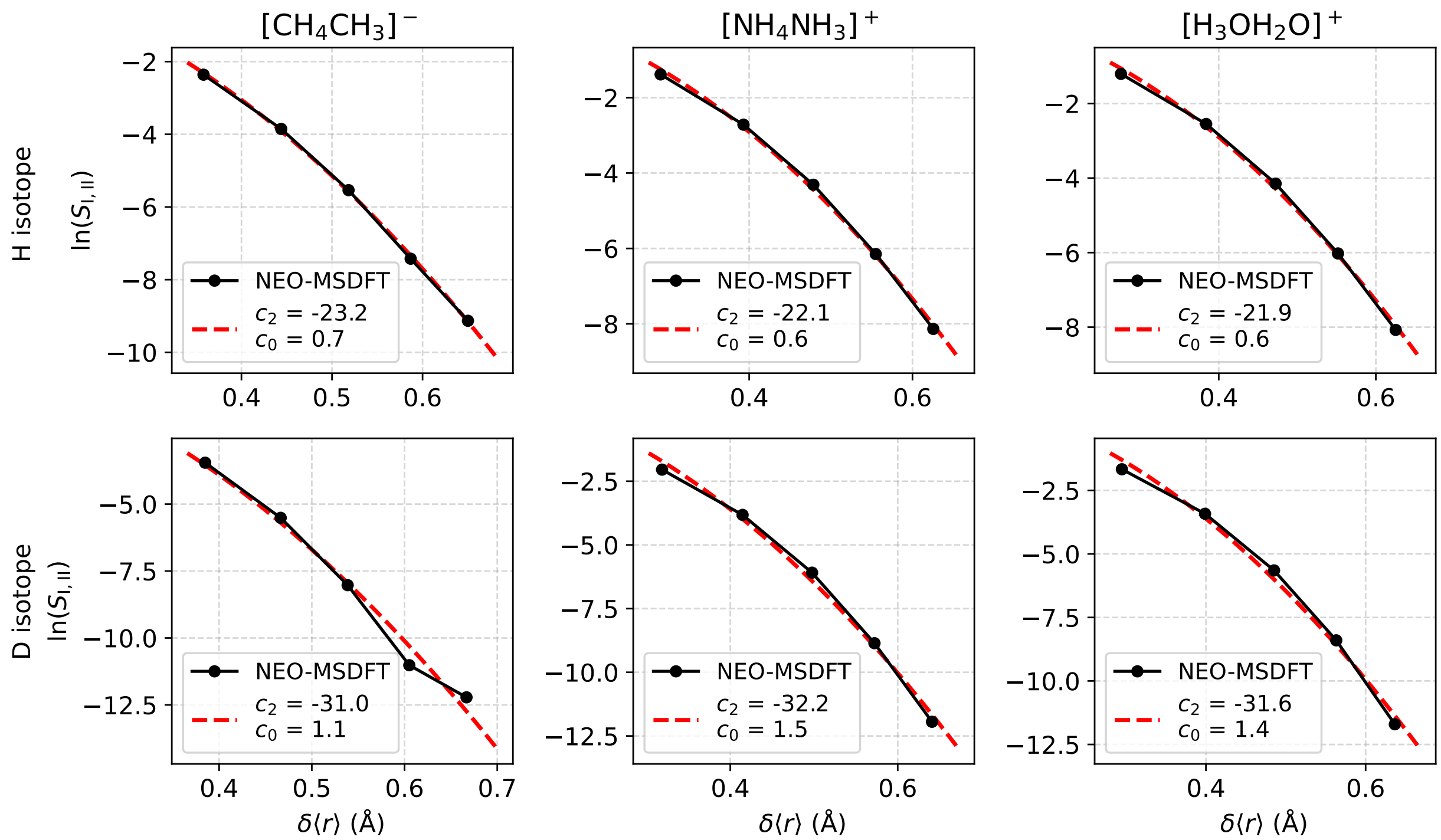

Figure S5. Natural logarithm of the overlap $S_{\text{I,II}}$ as a function of the distance between the proton position expectation values of the localized diabatic states, $\delta \langle r \rangle$, calculated with NEO-MSDFT. The computed data was fitted to $y = c_2 x^2 + c_0$. The resulting parameters are given in the legend.

approximate formula for the vibronic coupling given by eq S14 as a function of r:

$$V(r) = \frac{e^{-ar^2}}{1 - e^{-2ar^2}} \times br^2 = \frac{br^2}{2\sinh\left(ar^2\right)} \tag{S18}$$

For a symmetric system, the tunneling splitting $\Delta(r)$ is thus

$$\Delta(r) = 2V(r) = \frac{br^2}{\sinh\left(ar^2\right)} \tag{S19}$$

We can use this equation to fit the tunneling splittings calculated with both NEO-MSDFT and FGH. In this analysis, a is a fitting parameter that quantifies the width of the localized wavefunctions and b is a fitting parameter related to the energy of the off-diagonal Hamiltonian element.

For this analysis, we only consider the H isotope data. The calculated tunneling splittings

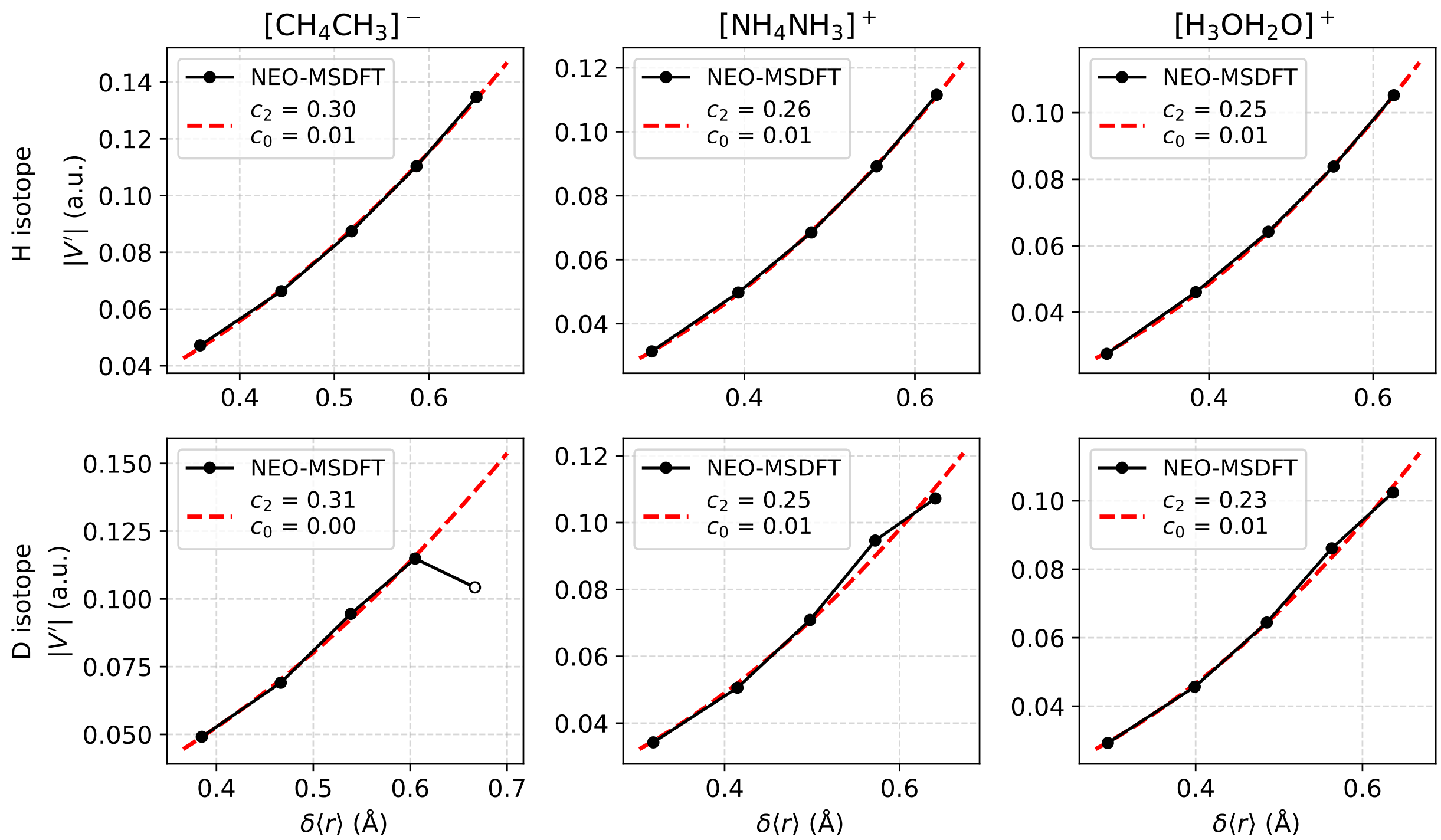

Figure S6. $|V'|$, given by eq S14, as a function of the distance between the proton position expectation values of the localized diabatic states, $\delta\langle r\rangle$, calculated with NEO-MSDFT. The computed data was fitted to $y = c_2x^2 + c_0$. The resulting parameters are given in the legend. The longest distance point for the D isotope of $[CH_4CH_3]^-$ was not included in the fitting, as indicated by the unfilled circle.

and fits are shown in Figure S7 with the parameters given in the legends. For the FGH data, the $\delta\langle r\rangle$ values were calculated by first localizing the wavefunctions by taking the in-phase and out-of-phase combinations of the two lowest energy wavefunctions and calculating the proton position expectation values by numerical integration. The simple model defined in eq S19 fits the data reasonably well, suggesting that the fitting parameters can be compared and interpreted.

First, we examine the a parameter. For NEO-MSDFT, its value is between 22 and 24 Å^{-2} for all three molecules, similarly to what was found in Figure S5. For FGH, the value of the a parameter is between 13 and 15 Å^{-2}. This trend supports the earlier hypothesis that the NEO-DFT densities are still overly localized. Numerically, these results indicate that to reproduce the FGH results with the functional form for $\Delta(r)$ fit to the NEO-MSDFT results,

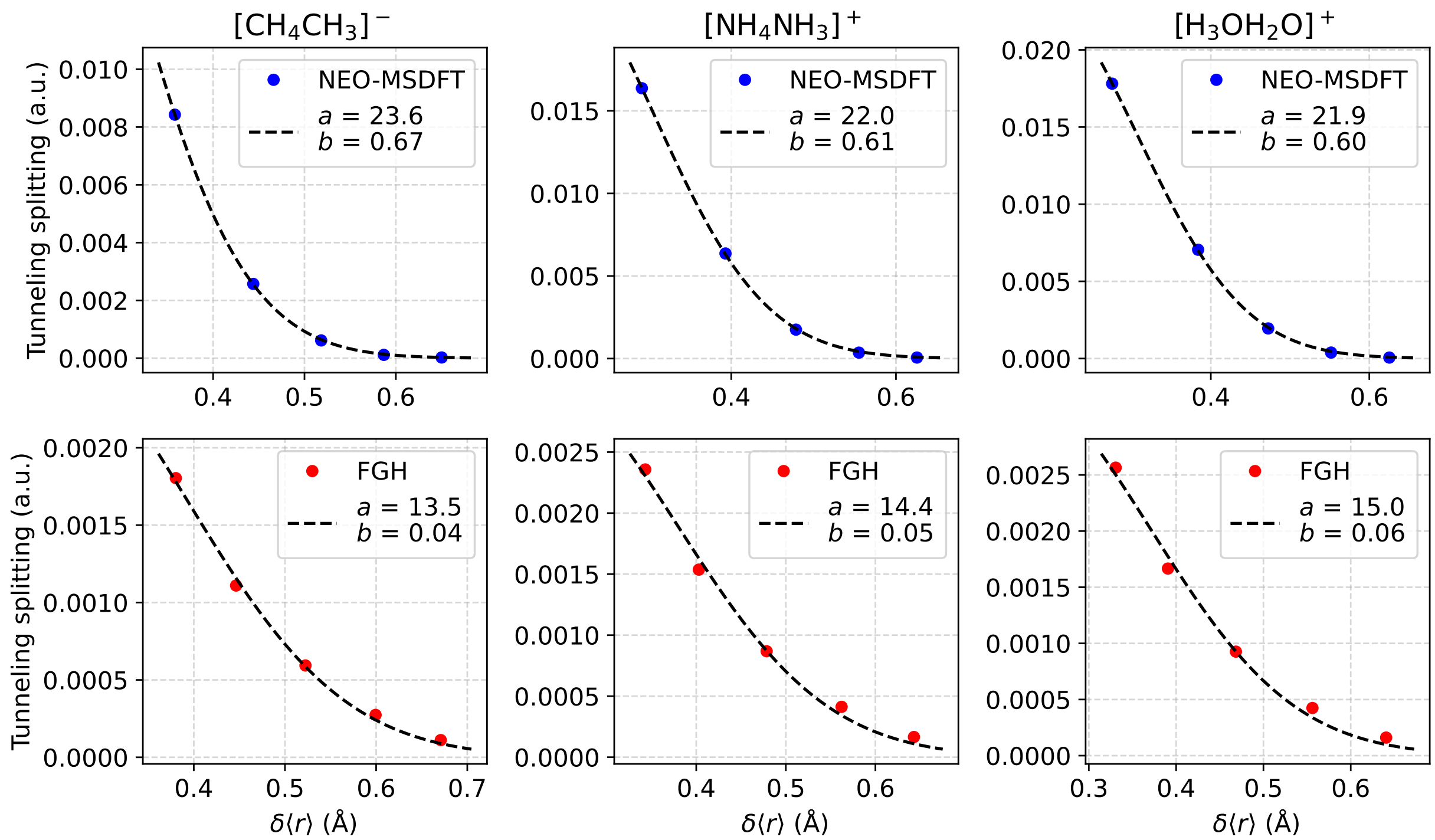

Figure S7. H isotope tunneling splitting as a function of the distance between the proton position expectation values of the localized diabatic states, $\delta \langle r \rangle$. The data was fitted to the expression in eq S19. The resulting parameters are given in the legend. The units of a are Å^{-2}, and the units of b are E_hÅ^{-2}.

we should multiply a^{MSDFT} by $\beta = a^{\mathrm{FGH}}/a^{\mathrm{MSDFT}} \approx 0.6$, where the exact factor is somewhat system-dependent. Considering our approximation that $S_{\mathrm{I,II}} = e^{-ar^2}$, this finding implies that the corrected overlap should be $S'_{\mathrm{I,II}} = S_{\mathrm{I,II}}^{\beta}$. The β value found here is reasonably close to the value of 0.492 from the original paper.[13]

Next, we analyze the b parameter. The ratio $\alpha = b^{\mathrm{FGH}}/b^{\mathrm{MSDFT}}$ ranges from 0.06 to 0.1. This range includes 0.0604, which is the α that was found in the original paper as a multiplicative constant to the overlap (eq S15). The analysis based on eq S19 suggests that α should multiply the $|V'|$ term instead. This analysis leads to the scaling scheme given in eq S17.

S4 Comparison of NEO-DFT and CNEO-DFT Optimized Geometries

Table S2 shows the difference in the proton position expectation values and energies from constrained geometry optimizations of the HCN molecule, where the CN distance was fixed to 1.1 Å and only the proton basis function center position was optimized. The NEO-DFT energies are lower than the CNEO-DFT energies by 10^{-4} Hartree at most. The proton position expectation values differ by around 10^{-4} Å.

Table S2. Comparison of NEO-DFT and CNEO-DFT Geometry Optimizations of the HCN Molecule [a]

Nuc. basis set	El. basis set	$\langle r \rangle_{\mathrm{CNEO}} - \langle r \rangle_{\mathrm{NEO}}$ [b]	$E_{\mathrm{CNEO}} - E_{\mathrm{NEO}}$ [c]
PB4-F2	def2-SVPD	6.3×10^{-4}	1.0×10^{-4}
PB4-F2	def2-TZVPD	8.7×10^{-5}	8.2×10^{-5}
PB4-F2	def2-QZVPD	-1.1×10^{-4}	3.9×10^{-6}
PB5-G	def2-SVPD	7.8×10^{-4}	1.0×10^{-4}
PB5-G	def2-TZVPD	1.5×10^{-4}	8.0×10^{-5}
PB5-G	def2-QZVPD	-1.7×10^{-5}	8.7×10^{-8}

[a] These calculations were performed with the ωB97X and epc17-2 functionals.
[b] Difference in the proton position expectation values along the C-H bond in Å.
[c] Difference in the NEO-SCF energies in Hartree.

S5 Critical Distance Estimation

The critical distance R_{c} is defined as the donor–acceptor distance at which the corrected overlap, $S'_{ab}(R) = S^{\beta}_{ab}(R)$, approaches unity. To determine R_{c}, the corrected overlap values were fitted to the functional form

$$S'_{ab}(R) = S_0 \exp\left[c_1 R + c_2 R^2\right] \tag{S20}$$

and R_{c} is determined by the condition $S'_{ab}(R_{\mathrm{c}}) = 1$.

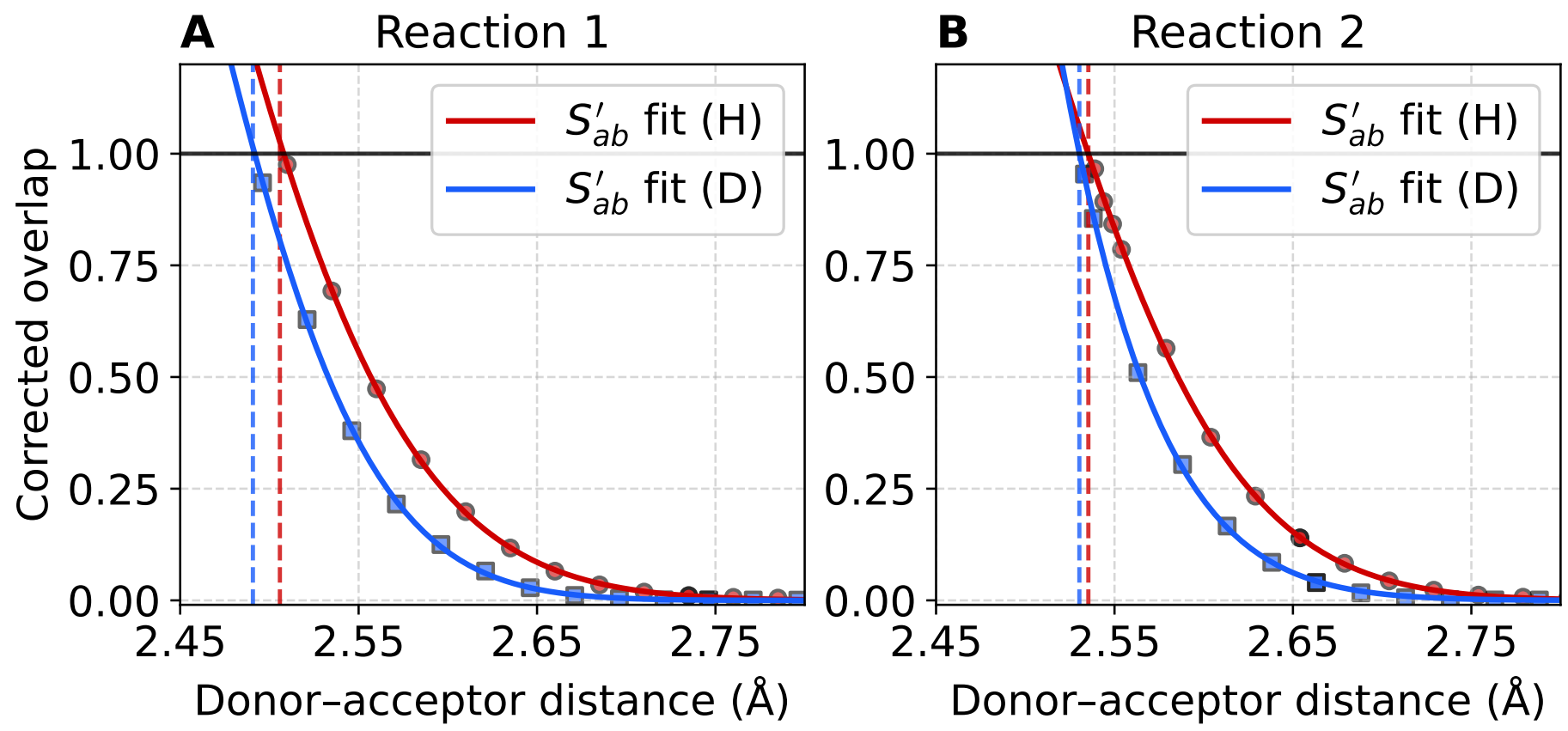

Figure S8. Corrected overlap as a function of the donor–acceptor distance for Reactions 1 (A) and 2 (B). The vertical dashed lines indicate the critical distance for the H isotope (red) and D isotope (blue).

S6 Parameters for Fitting Functions

The vibronic reactant and MECP energies as functions of the proton donor–acceptor distance R were fit to a fourth-order polynomial, subject to the constraint that the function remains convex for all values of R. The vibronic couplings as functions of R were fit to the functional form given in eq 26 from the main text. The fixed parameters E_0 and R_0 are given in Table S3, and the fitting parameters are given in Tables S4 to S6.

Table S3. Fixed Parameters E_0 and R_0 for $V_{\mathrm{r}}(R)$ and $V_{\mathrm{MECP}}(R)$ (in a.u.)

Reaction	Surface	$E_0{}^a$	R_0
**1**-H	V_{r}	0	5.228
	V_{MECP}	3.705×10^{-4}	5.168
**1**-D	V_{r}	0	5.258
	V_{MECP}	5.565×10^{-4}	5.189
**2**-H	V_{r}	0	5.100
	V_{MECP}	3.938×10^{-4}	5.015
**2**-D	V_{r}	0	5.134
	V_{MECP}	6.458×10^{-4}	5.033

[a] E_0 for V_{r} is set to zero for each reaction, and E_0 for V_{MECP} is reported relative to that for V_{r}.

Table S4. Fitting Parameters for $V_{ab}(R)$ (in a.u.)

	$V_{ab}(R) = V_0 \exp[c_1 R + c_2 R^2]$		
Reaction	V_0	c_1	c_2
**1**-H	7.499×10^{-82}	7.956×10^{1}	-8.750×10^{0}
**1**-D	2.454×10^{-92}	8.858×10^{1}	-9.573×10^{0}
**2**-H	3.772×10^{-136}	1.334×10^{2}	-1.457×10^{1}
**2**-D	1.902×10^{-131}	1.280×10^{2}	-1.389×10^{1}

Table S5. Fitting Parameters[a] for $V_{\mathrm{r}}(R)$ (in a.u.)

	$V_{\mathrm{r}}(R) = E_0 + \sum_{i=1}^{4} a_i (R - R_0)^i$			
Reaction	a_1	a_2	a_3	a_4
**1**-H	-6.059×10^{-5}	1.155×10^{-2}	-5.581×10^{-3}	2.743×10^{-3}
**1**-D	-1.017×10^{-4}	1.149×10^{-2}	-5.990×10^{-3}	3.253×10^{-3}
**2**-H	-6.100×10^{-5}	1.365×10^{-2}	-5.381×10^{-3}	1.406×10^{-3}
**2**-D	-1.076×10^{-4}	1.399×10^{-2}	-6.113×10^{-3}	1.302×10^{-3}

[a] E_0 and R_0 are the energy and donor–acceptor distance, respectively, of the unconstrained optimized geometry and are therefore fixed quantities rather than fitting parameters. Their values are provided in Table S3.

Table S6. Fitting Parameters[a] for $V_{\mathrm{MECP}}(R)$ (in a.u.)

	$V_{\mathrm{MECP}}(R) = E_0 + \sum_{i=1}^{4} a_i (R - R_0)^i$			
Reaction	a_1	a_2	a_3	a_4
**1**-H	8.532×10^{-5}	1.344×10^{-2}	-8.725×10^{-3}	6.003×10^{-3}
**1**-D	1.879×10^{-5}	1.326×10^{-2}	-8.460×10^{-3}	5.839×10^{-3}
**2**-H	1.280×10^{-4}	1.772×10^{-2}	-1.263×10^{-2}	8.777×10^{-3}
**2**-D	-4.618×10^{-5}	1.745×10^{-2}	-1.153×10^{-2}	5.294×10^{-3}

[a] E_0 and R_0 are the energy and donor–acceptor distance, respectively, of the unconstrained optimized geometry and are therefore fixed quantities rather than fitting parameters. Their values are provided in Table S3.

S7 Numerical Results

Table S7. Calculated H/D KIE Values Computed with Different Methods at Varying Temperatures

	Reaction 1				Reaction 2			
T / K	TST	RPI[a]	NEO(LZ)[b]	NEO(WC)[c]	TST	RPI[a]	NEO(LZ)[b]	NEO(WC)[c]
300	3.9	–	9.1	12.2	3.8	–	5.2	8.0
275	4.4	–	10.0	13.4	4.2	–	5.4	8.4
250	5.0	–	11.1	14.8	4.8	–	5.7	9.0
225	5.9	–	12.6	16.7	5.7	–	6.1	9.7
200	7.3	–	14.7	19.1	7.0	–	6.5	10.6
175	9.6	11.5	17.6	22.5	9.3	–	7.2	11.8
150	13.8	19.0	21.9	27.3	13.4	8.1	8.2	13.5
125	23.2	27.8	29.0	34.7	22.3	11.5	9.7	15.8
100	50.2	44.4	41.9	47.0	48.4	16.6	12.4	19.3
75	183.2	60.5	70.4	69.8	175.6	24.8	18.0	24.9
50	2452.5	82.0	159.8	120.1	2320.9	33.9	35.0	34.4

[a] Missing data corresponds to temperatures above the crossover temperature.
[b] Computed using the Landau–Zener transmission probability.
[c] Computed using the weak coupling transmission probability.

S8 Sensitivity Analysis

Figure S9 shows the KIEs for both reaction, where both $\boldsymbol{g}_a$ and $\boldsymbol{g}_b$ were multiplied or divided by a factor of 5 (panels A and B) or μ was multiplied or divided by a factor of 5 (panels C and D). These results suggest that even if the gradient or reduced mass are considerably inaccurate because they are assumed to be independent of the proton donor–acceptor distance R, the qualitative behavior of the KIEs computed with NEO-GRT is still valid. However, we do not expect that assuming $\boldsymbol{g}_a$, $\boldsymbol{g}_b$, and μ to be independent of R is introducing errors as large as those shown in Figure S9, especially in the low-temperature regime, where a relatively small range of R values is contributing to the rate constant. In preliminary calculations for the reactions studied herein, we observed that μ changes by less than 5% and $\Delta\boldsymbol{g}$ changes by approximately a factor of two over the full range of relevant R values.

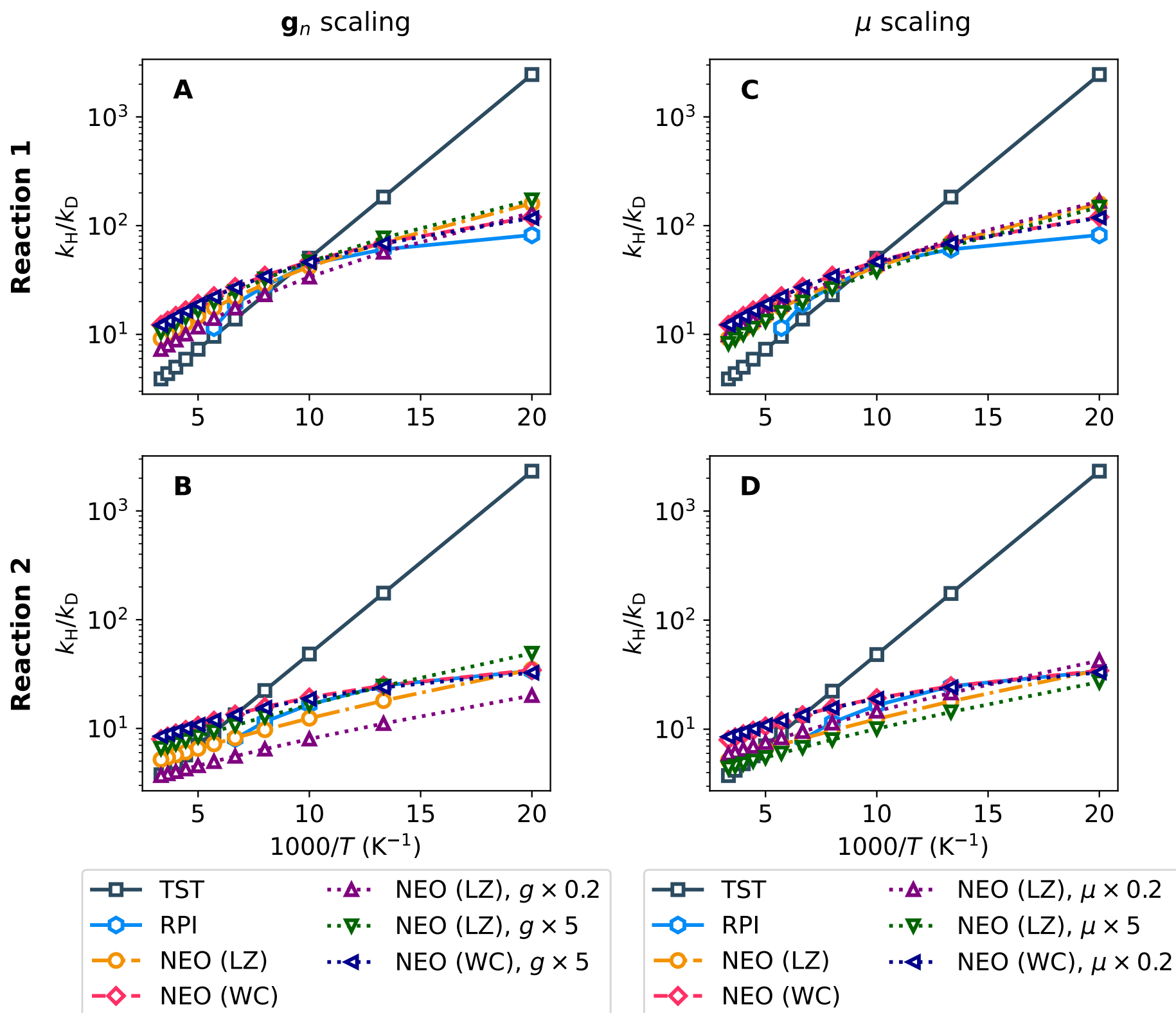

Figure S9. Sensitivity analysis for the gradient terms (A and B) and for the reduced mass (C and D).

S9 Detailed Balance and Reverse Reaction Analysis

S9.1 Detailed Balance

In this subsection, we show that NEO-GRT obeys detailed balance,

$$\frac{k_{a\to b}}{k_{b\to a}} = e^{-\beta(G_b - G_a)} = \frac{Q_b}{Q_a} \tag{S21}$$

where G_n is the Gibbs free energy at the minimum of diabatic potential n and Q_n is the respective partition function. We assume that $Q_\mathrm{r}(T,R)$ and $Q_\mathrm{MECP}(T,R)$ are constant with their values corresponding to those calculated at the equilibrium distances, i.e., $Q_\mathrm{r}(T) \equiv$

$Q_\mathrm{r}(T, R_\mathrm{r}^\mathrm{eq})$ and $Q_\mathrm{MECP}(T) \equiv Q_\mathrm{MECP}(T, R_\mathrm{MECP}^\mathrm{eq})$. In this case, the rate constant expression is

$$k_{a\to b}(T) = \int_0^\infty P_a(T,R)\, k_{a\to b}(T,R)\, \mathrm{d}R \tag{S22}$$

$$P_a(T,R) = \frac{e^{-\beta V_a(R)}}{\int_0^\infty e^{-\beta V_a(R')}\,\mathrm{d}R'} = \frac{e^{-\beta V_a(R)}}{Z_a(T)} \tag{S23}$$

$$k_{a\to b}(T,R) = \frac{1}{\beta h}\frac{Q^\ddagger(T)}{Q_a(T)} \times e^{-\beta V^\ddagger_{a\to b}(R)} \times \kappa(T,R) \tag{S24}$$

Considering that $V^\ddagger_{a\to b}(R) = V_\mathrm{MECP}(R) - V_a(R)$, we can rewrite the canonical rate constant expression as

$$k_{a\to b}(T) = \int_0^\infty \frac{e^{-\beta V_a(R)}}{Z_a(T)}\frac{1}{\beta h}\frac{Q^\ddagger(T)}{Q_a(T)} \times e^{-\beta V^\ddagger_{a\to b}(R)} \times \kappa(T,R)\,\mathrm{d}R \tag{S25a}$$

$$= \frac{1}{\beta h}\frac{Q^\ddagger(T)}{Z_a(T)Q_a(T)}\int_0^\infty e^{-\beta V_\mathrm{MECP}(R)} \times \kappa(T,R)\,\mathrm{d}R \tag{S25b}$$

As $e^{-\beta V_\mathrm{MECP}(R)}$ and $\kappa(T,R)$ are equal for both the forward and backward reactions, at least for the Landau–Zener and weak coupling κ formulations used in this work, the integral in eq S25b is equal for the forward and reverse reactions. The ratio of the forward and reverse reaction rate constants is then

$$\frac{k_{a\to b}(T)}{k_{b\to a}(T)} = \frac{\frac{1}{\beta h}\frac{Q^\ddagger(T)}{Z_a(T)Q_a(T)}\int_0^\infty e^{-\beta V_\mathrm{MECP}(R)} \times \kappa(T,R)\,\mathrm{d}R}{\frac{1}{\beta h}\frac{Q^\ddagger(T)}{Z_b(T)Q_b(T)}\int_0^\infty e^{-\beta V_\mathrm{MECP}(R)} \times \kappa(T,R)\,\mathrm{d}R} = \frac{Z_b(T)Q_b(T)}{Z_a(T)Q_a(T)} \tag{S26}$$

where $Z(T)Q(T)$ is the product of the partition function associated with coordinate R and the partition function associated with the rest of the modes, constituting the total partition function within the approximations underlying the separate treatment of the donor–acceptor distance R. Thus, the ratio corresponds to the detailed balance result of eq S21,

$$\frac{k_{a\to b}(T)}{k_{b\to a}(T)} = \frac{Q_b^\mathrm{tot}(T)}{Q_a^\mathrm{tot}(T)} = e^{-\beta(G_b(T)-G_a(T))} \tag{S27}$$

S9.2 Results for Reverse Reactions

We calculated the KIEs for the reverse reactions with the TST, RPI, and NEO-GRT methods. For the reverse reactions, NEO-GRT does not agree as well with RPI theory (compare blue and red curves in Figure S10). To understand the origin of this discrepancy, we also calculated the KIEs for the reverse reactions via detailed balance,

$$\mathrm{KIE}_{b\to a} = \mathrm{KIE}_{a\to b} \times e^{\beta\Delta\Delta G} \tag{S28}$$

where $\Delta\Delta G = (G_b^{\mathrm{H}} - G_a^{\mathrm{H}}) - (G_b^{\mathrm{D}} - G_a^{\mathrm{D}})$. Here the a and b subscripts denote the reactant and product, respectively, shown in Scheme 1 in the main text.

We calculated the free energies of the reactants and products with both conventional DFT and CNEO-DFT. For conventional DFT, we calculated the free energy by computing the ZPE and vibrational entropic contributions using the normal modes based on the harmonic oscillator model. For CNEO-DFT, we calculated the free energy using the scheme previously used for pK_a calculations,[30] where the ZPE associated with the quantum nuclei is included directly within the CNEO-DFT energy, and the ZPE associated with the other nuclei is computed using the normal modes obtained from the reduced Hessian $\mathbf{H}_n$ defined by eq 20 in the main text. In this scheme, the vibrational entropic terms are computed using the normal modes from the extended CNEO-DFT Hessian, $\mathbf{H}_n^{\mathrm{ext}}$, defined in the main text. The rotational and translational entropic contributions were also included for both the conventional DFT and CNEO-DFT calculations using the standard models.

We then used these free energies to calculate the KIEs for the reverse reaction from the KIEs for the forward reaction according to eq S28. Note that this type of calculation is not expected to satisfy detailed balance exactly for the NEO-GRT method because the $\Delta\Delta G$ term calculated from the Hessians at the minima does not exactly correspond to the partition function definitions in eq S26. However, we found that the KIEs for the reverse reactions calculated directly with NEO-GRT or calculated from the KIEs of the forward reaction via

eq S28 using the CNEO-DFT reaction free energies are in excellent agreement (compare red and purple curves in Figure S10). This agreement provides further validation for the NEO-GRT approach and the procedures used to compute the input quantities. As expected, the KIEs for the reverse reactions calculated directly with RPI theory or calculated from the KIEs of the forward reaction via eq S28 using the conventional NEO-DFT reaction free energies are in perfect agreement (compare blue and navy curves in Figure S10). Thus, both approaches satisfy detailed balance.

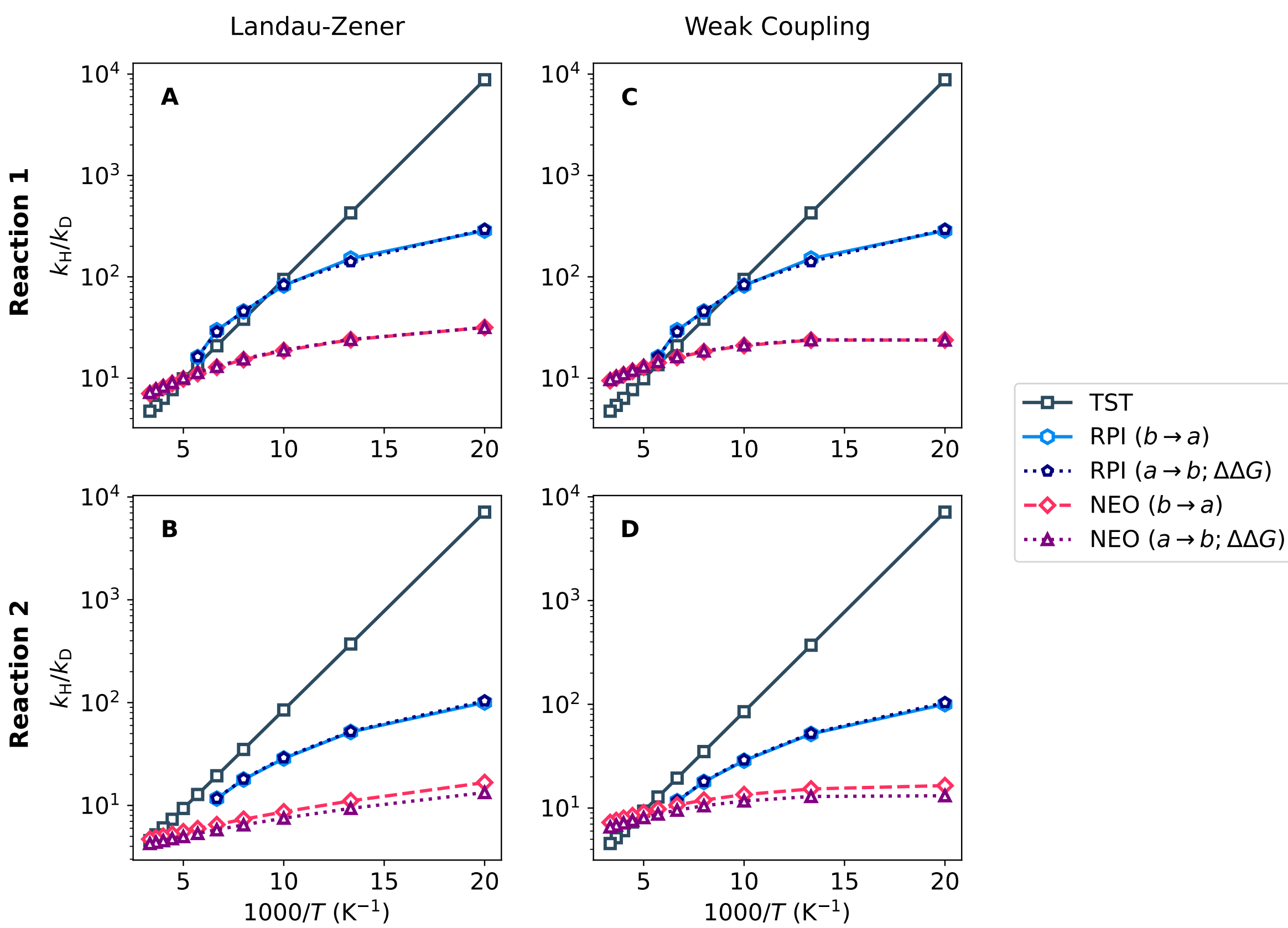

Figure S10. KIEs calculated for the reverse reactions with TST, RPI theory, and NEO-GRT. (A,B) NEO-GRT KIEs obtained using the LZ transmission probability for Reaction 1 (A) and 2 (B). (C,D) NEO-GRT KIEs obtained using the WC transmission probability for Reaction 1 (C) and 2 (D). RPI($b \to a$) corresponds to the KIEs calculated for the reverse reaction directly with RPI theory. RPI($a \to b$; $\Delta\Delta G$) corresponds to the KIEs calculated from the RPI forward reaction KIEs via detailed balance using free energies from conventional DFT. NEO($b \to a$) corresponds to the KIEs calculated for the reverse reaction directly with NEO-GRT. NEO($a \to b$; $\Delta\Delta G$) corresponds to the KIEs calculated from the NEO-GRT forward reaction KIEs via detailed balance using free energies from CNEO-DFT.

Upon further analysis, we determined that the discrepancy between the NEO-GRT and RPI KIEs for the reverse reactions is due mainly to small differences in the reaction free energies, specifically the ZPEs, computed with conventional DFT and CNEO-DFT (see Table S8). At low temperatures, a small difference on the order of 0.2 kcal/mol in $\Delta\Delta G$ can lead to an order of magnitude difference in the KIE. One hypothesis is that anharmonicity is causing the differences between the relative free energies used to compute the NEO-GRT and RPI KIEs. The free energies calculated with conventional DFT, as well as our implementation of RPI theory, rely fully on the harmonic approximation, whereas the free energies calculated with CNEO-DFT and NEO-GRT include the anharmonic ZPE associated with the transferring hydrogen. This anharmonicity has been shown to be important for hydrogen-bonded systems,[30] such as the intramolecular proton transfer systems studied herein.

Another hypothesis is that the differences between the NEO-GRT and RPI KIEs are due to the approximate epc17-2 functional, which provides a reasonable but not complete description of electron–proton correlation and therefore could introduce relatively small errors in the ZPEs. To test this hypothesis, we performed NEO coupled-cluster singles and doubles with perturbative triples (NEO-CCSD(T))[31–33] calculations on the geometries optimized with CNEO-DFT for the reactant, MECP, and product at a series of different R values. We applied the following correction to the CNEO-DFT energies:

$$\Delta\text{ZPE}_{\text{NEO-CCSD(T)}}(R) = E_{\text{NEO-CCSD(T)}}(R) - E_{\text{CCSD(T)}}(R) - E_{\text{CNEO-DFT}}(R) + E_{\text{DFT}}(R) \quad \text{(S29)}$$

where $E_{\text{NEO-CCSD(T)}}(R)$, $E_{\text{CCSD(T)}}(R)$, $E_{\text{CNEO-DFT}}(R)$, and $E_{\text{DFT}}(R)$ are the NEO-CCSD(T), conventional CCSD(T), CNEO-DFT, and conventional DFT energies, respectively, evaluated at the CNEO-DFT optimized geometry for a given R.

The reaction free energies obtained after applying this correction are provided in Table S8. We also applied this NEO-CCSD(T) ZPE correction to $V_{\text{r}}(R)$ and $V_{\text{MECP}}(R)$. For

the NEO-CCSD(T) calculations, the aug-cc-pVDZ electronic basis set was used for all atoms excluding the transferring hydrogen, and the aug-cc-pV6Z electronic basis set was used for the transferring hydrogen, along with the PB4-F2 protonic basis set. The same electronic basis sets were used for the conventional CCSD(T) calculations. Note that these electronic basis sets may not be fully converged for the absolute NEO-CCSD(T) ZPEs but appear to provide accurate relative NEO-CCSD(T) ZPEs. Note also that after applying the NEO-CCSD(T) ZPE corrections, $V_{\mathrm{MECP}}(R)$ differs slightly for the two diabatic vibronic states, and therefore we used an average of these two energies for the KIE calculations.

The KIEs obtained with NEO-GRT using the NEO-CCSD(T)-corrected ZPEs for the forward and reverse reactions are shown in Figure S11. Interestingly, the resulting NEO-GRT KIEs agree much better with the RPI KIEs for the reverse reaction and still agree well with the RPI KIEs for the forward reaction. This improvement suggests that the discrepancy between the RPI and NEO-GRT KIEs for the reverse reactions is due mainly to small errors in the ZPEs computed with the epc17-2 functional. Such errors can be corrected by single-point NEO-CCSD(T) calculations. In some cases, such as the forward reactions, this type of correction is not necessary. The remaining minor discrepancies may be due to the neglect of anharmonicity in the RPI calculations or to other approximations underlying the NEO-GRT or RPI method. Importantly, at higher temperatures, such small errors in the ZPEs will have a much less significant impact on the KIEs.

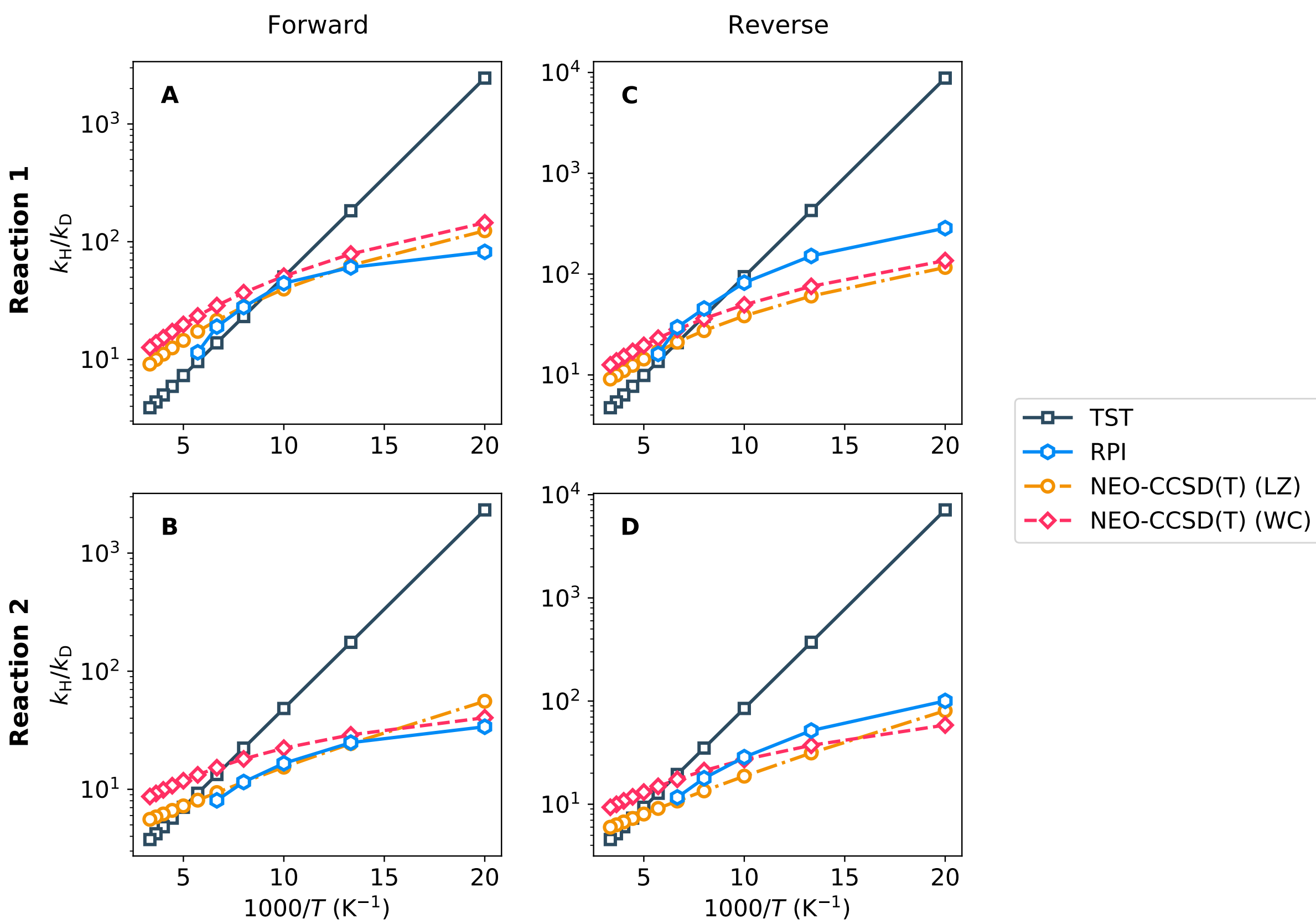

Figure S11. KIEs calculated for the forward and reverse reactions with TST, RPI theory, and NEO-GRT. (A,B) KIEs for the forward direction for Reaction 1 (A) and 2 (B). (C,D) KIEs for the reverse direction for Reaction 1 (C) and 2 (D). NEO-CCSD(T) corresponds to the KIEs calculated with the NEO-GRT method, where $V_r(R)$ and $V_{MECP}(R)$ were corrected with single-point NEO-CCSD(T) calculations using eq S29, as described in the text.

Table S8. Reaction Free Energies for the Forward Reactions (in kcal/mol)

	CNEO-DFT			DFT			NEO-CCSD(T)[a]		
T / K	ΔG^{H}	ΔG^{D}	$\Delta\Delta G$	ΔG^{H}	ΔG^{D}	$\Delta\Delta G$	ΔG^{H}	ΔG^{D}	$\Delta\Delta G$
Reaction 1									
0[b]	-0.99	-0.83	-0.16	0.12	-0.01	0.13	0.09	0.10	-0.01
50	-1.00	-0.84	-0.16	0.11	-0.02	0.13	0.08	0.09	-0.01
75	-1.00	-0.85	-0.16	0.10	-0.03	0.13	0.07	0.08	-0.01
100	-1.01	-0.85	-0.16	0.09	-0.03	0.12	0.06	0.07	-0.01
125	-1.02	-0.86	-0.16	0.09	-0.03	0.12	0.06	0.07	-0.01
150	-1.02	-0.87	-0.15	0.09	-0.03	0.12	0.05	0.06	-0.01
175	-1.03	-0.88	-0.15	0.09	-0.03	0.12	0.05	0.05	-0.003
200	-1.03	-0.88	-0.15	0.09	-0.03	0.12	0.04	0.05	-0.002
225	-1.03	-0.89	-0.15	0.09	-0.03	0.12	0.04	0.04	0.0004
250	-1.04	-0.89	-0.14	0.09	-0.03	0.12	0.04	0.04	0.003
275	-1.04	-0.90	-0.14	0.09	-0.03	0.12	0.04	0.03	0.005
300	-1.04	-0.90	-0.14	0.09	-0.03	0.12	0.03	0.02	0.01
Reaction 2									
0[b]	-2.31	-2.22	-0.09	-1.32	-1.44	0.12	-1.46	-1.46	0.004
50	-2.30	-2.21	-0.10	-1.30	-1.42	0.12	-1.45	-1.45	0.002
75	-2.29	-2.20	-0.10	-1.28	-1.40	0.12	-1.44	-1.44	0.00005
100	-2.28	-2.18	-0.10	-1.26	-1.37	0.11	-1.43	-1.43	-0.002
125	-2.27	-2.17	-0.10	-1.24	-1.35	0.11	-1.42	-1.41	-0.004
150	-2.26	-2.15	-0.10	-1.21	-1.32	0.11	-1.40	-1.40	-0.01
175	-2.24	-2.14	-0.10	-1.18	-1.30	0.12	-1.39	-1.38	-0.01
200	-2.23	-2.12	-0.11	-1.16	-1.27	0.11	-1.37	-1.36	-0.01
225	-2.21	-2.10	-0.11	-1.12	-1.24	0.12	-1.36	-1.35	-0.01
250	-2.20	-2.08	-0.11	-1.09	-1.21	0.12	-1.34	-1.33	-0.01
275	-2.18	-2.07	-0.11	-1.06	-1.17	0.11	-1.32	-1.31	-0.02
300	-2.16	-2.05	-0.12	-1.03	-1.14	0.11	-1.31	-1.29	-0.02

[a] G values computed in the same manner as with CNEO-DFT, except that the ZPE associated with the quantum nucleus is corrected at the NEO-CCSD(T) level of theory using eq S29.
[b] G values at 0 K correspond to $V + \mathrm{ZPE}$.

S10 RPI Additional Computational Details

This subsection provides additional computational details for our ring-polymer instanton (RPI) calculations. We followed the protocol explained in ref 34 and used in-house Python codes provided by Jeremy Richardson (ETH Zurich). In brief, we optimized the reactant and transition state (TS) geometries and performed normal-mode analyses on these geometries. We used the imaginary mode of the TS to calculate the crossover temperature as

$$T_c = \frac{\hbar c \tilde{\nu}}{k_{\mathrm{B}}} \tag{S30}$$

where $\hbar$ is the reduced Planck's constant, c is the speed of light, k_{B} is the Boltzmann constant, and $\tilde{\nu}$ is the frequency in wavenumbers associated with the imaginary mode at the TS. The frequencies and crossover temperatures are given in Table S9. The highest temperature for RPI optimizations for each case was selected to be at least 20 K below the crossover temperature. For each temperature, the number of beads N was selected to be the lowest power of 2 satisfying $N > 40 \times T_{\mathrm{c}}/T$, which has been shown to produce errors below 10%.[35] The bead counts for all calculations are given in Table S10. Note that because the instanton trajectory folds back on itself,[34] we only need to optimize half of the ring-polymer, e.g., a 128-bead RPI calculation requires the optimization of a 64-bead ring-polymer. The half-ring-polymer potential is

$$U_{N/2}(\boldsymbol{x}) = \sum_{i=1}^{N/2-1} \frac{1}{2\beta_N^2\hbar^2}|x_{i+1} - x_i|^2 + \sum_{i=1}^{N/2} V(x_i) \tag{S31}$$

where $\beta_N = \beta/N$, x_i are mass-weighted coordinates of bead i, and $V(x_i)$ is the potential energy evaluated at bead i. The instanton trajectory corresponds to a first-order saddle point of $U_{N/2}(\boldsymbol{x})$, and the action of the full instanton is $S = 2\hbar\beta_N U_{N/2}(\boldsymbol{x})$ evaluated at this saddle point.

In each case, the initial RPI guess with 16 beads was generated by displacements along

the imaginary mode at the TS. This guess was used for the optimization of the highest-temperature RPI with 16 beads, followed by optimizations with 32 beads and 64 beads. Each subsequent RPI optimization at the next lower temperature started from the previous optimized result as the initial guess, using interpolation when increasing the number of beads was required (see Table S10). For each optimized RPI, Hessians were calculated at each bead to build the ring-polymer Hessian for the rate constant calculation.

The rate constant was calculated according to the RPI rate theory formula as

$$k_{\mathrm{RPI}} = \frac{1}{2\pi\beta\hbar}\frac{Z_{\mathrm{RPI}}}{Z_r}e^{-S/\hbar} \tag{S32}$$

where Z_{RPI} and Z_{r} are the partition functions of the RPI and the reactant, respectively, and S is the instanton action. The vibrational component of the RPI partition function was calculated from the eigenvalues of the ring-polymer Hessian, and the rotational and translational components were calculated with the classical expressions adapted to the ring-polymer formalism.[34] The reactant partition function was calculated by analogous expressions for a collapsed ring-polymer, where each bead had the geometry of the reactant.

Table S9. Transition State Imaginary Mode Frequency and Crossover Temperature

	Reaction 1		Reaction 2	
	H	D	H	D
$\tilde{\nu}$ [a] / cm^{-1}	1202.3	877.1	1059.9	781.3
T_{c} [b] / K	275.3	200.8	242.7	178.9

[a] Frequency in wavenumbers associated with the imaginary mode at the TS.
[b] Crossover temperature calculated according to eq S30.

Table S10. Number of Beads in RPI Calculations[a]

	Reaction 1		Reaction 2	
T / K	H	D	H	D
250	64	–	–	–
225	64	–	64	–
200	64	–	64	–
175	64	64	64	–
150	128	64	128	64
125	128	128	128	64
100	128	128	128	128
75	256	128	256	128
50	256	256	256	256

[a] Number of beads N was selected to be the lowest power of 2 satisfying $N > 40 \times T_c/T$.

References

(1) Lykhin, A. O.; Kaliakin, D. S.; dePolo, G. E.; Kuzubov, A. A.; Varganov, S. A. Nonadiabatic Transition State Theory: Application to Intersystem Crossings in the Active Sites of Metal-Sulfur Proteins. *Int. J. Quantum Chem.* **2016**, *116*, 751–761.

(2) Dergachev, V. D.; Rooein, M.; Dergachev, I. D.; Lykhin, A. O.; Mauban, R. C.; Varganov, S. A. NAST: Nonadiabatic Statistical Theory Package for Predicting Kinetics of Spin-Dependent Processes. *Top. Curr. Chem.* **2022**, *380*, 15–23.

(3) Nitzan, A. *Chemical Dynamics in Condensed Phases: Relaxation, Transfer and Reactions in Condensed Molecular Systems*; Oxford Graduate Texts; Oxford University Press, 2006.

(4) Backstrom, N.; Burton, N. A.; Turega, S.; Watt, C. I. F. The Primary Kinetic Hydrogen Isotope Effect in the Deprotonation of a Nitroalkane by an Intramolecular Carboxylate Group. *J. Phys. Org. Chem.* **2008**, *21*, 603–613.

(5) Backstrom, N.; Burton, N. A.; Watt, C. I. F. Primary Kinetic Hydrogen Isotope Effects

in Deprotonations of a Nitroalkane by Intramolecular Phenolate Groups. *J. Phys. Org. Chem.* **2010**, *23*, 711–722.

(6) Funderburk, L.; Lewis, E. S. Tunneling in a Proton Transfer. A Large Isotope Effect. *J. Am. Chem. Soc.* **1964**, *86*, 2531–2532.

(7) Bell, R. P.; Goodall, D. M. Kinetic Hydrogen Isotope Effects in the Ionization of Some Nitroparaffins. *Proc. R. Soc. Lond., A. Math. Phys. Sci.* **1966**, *294*, 273–297.

(8) Bordwell, F. G.; Boyle, W. J. Kinetic Isotope Effects for Nitroalkanes and Their Relation to Transition-State Structure in Proton-Transfer Reactions. *J. Am. Chem. Soc.* **1975**, *97*, 3447–3452.

(9) Caldin, E. F.; Mateo, S. Kinetic Isotope Effects and Tunnelling in the Proton-Transfer Reaction between 4-Nitrophenylnitromethane and Tetramethylguanidine in Various Aprotic Solvents. *J. Chem. Soc. Faraday Trans. 1: Phys. Chem. Condens. Ph.* **1975**, *71*, 1876.

(10) Caldin, E. F.; Mateo, S. Kinetic Isotope Effects and Tunnelling Corrections in the Proton-Transfer Reactions between 4-Nitrophenylnitromethane and Some Tertiary Amines in Aprotic Solvents. *J. Chem. Soc. Faraday Trans. 1: Phys. Chem. Condens. Ph.* **1976**, *72*, 112–121.

(11) Watt, C. I. F. Primary Kinetic Hydrogen Isotope Effects in Deprotonations of Carbon Acids. *J. Phys. Org. Chem.* **2010**, *23*, 561–571.

(12) Jarczewski, A.; Hubbard, C. D. A Review of Proton Transfer Reactions between Various Carbon-Acids and Amine Bases in Aprotic Solvents. *J. Mol. Struct.* **2003**, *649*, 287–307.

(13) Yu, Q.; Hammes-Schiffer, S. Nuclear-Electronic Orbital Multistate Density Functional Theory. *J. Phys. Chem. Lett.* **2020**, *11*, 10106–10113.

(14) Brorsen, K. R.; Yang, Y.; Hammes-Schiffer, S. Multicomponent Density Functional Theory: Impact of Nuclear Quantum Effects on Proton Affinities and Geometries. *J. Phys. Chem. Lett.* **2017**, *8*, 3488–3493.

(15) Yang, Y.; Brorsen, K. R.; Culpitt, T.; Pak, M. V.; Hammes-Schiffer, S. Development of a Practical Multicomponent Density Functional for Electron-Proton Correlation to Produce Accurate Proton Densities. *J. Chem. Phys.* **2017**, *147*, 114113.

(16) Lee, C.; Yang, W.; Parr, R. G. Development of the Colle-Salvetti Correlation-Energy Formula into a Functional of the Electron Density. *Phys. Rev. B* **1988**, *37*, 785.

(17) Becke, A. D. Density-Functional Thermochemistry. III. The Role of Exact Exchange. *J. Chem. Phys.* **1993**, *98*, 5648–5652.

(18) Stephens, P. J.; Devlin, F. J.; Chabalowski, C. F.; Frisch, M. J. Ab Initio Calculation of Vibrational Absorption and Circular Dichroism Spectra Using Density Functional Force Fields. *J. Phys. Chem.* **1994**, *98*, 11623–11627.

(19) Kendall, R. A.; Dunning, T. H.; Harrison, R. J. Electron Affinities of the First-Row Atoms Revisited. Systematic Basis Sets and Wave Functions. *J. Chem. Phys.* **1992**, *96*, 6796–6806.

(20) Woon, D. E.; Dunning, T. H. Gaussian Basis Sets for Use in Correlated Molecular Calculations. IV. Calculation of Static Electrical Response Properties. *J. Chem. Phys.* **1994**, *100*, 2975–2988.

(21) Yu, Q.; Pavošević, F.; Hammes-Schiffer, S. Development of Nuclear Basis Sets for Multicomponent Quantum Chemistry Methods. *J. Chem. Phys.* **2020**, *152*, 244123.

(22) Dickinson, J. A.; Yu, Q.; Hammes-Schiffer, S. Generalized Nuclear-Electronic Orbital Multistate Density Functional Theory for Multiple Proton Transfer Processes. *J. Phys. Chem. Lett.* **2023**, *14*, 6171–6178.

(23) Chai, J.-D.; Head-Gordon, M. Systematic Optimization of Long-Range Corrected Hybrid Density Functionals. *J. Chem. Phys.* **2008**, *128*, 84106.

(24) Knowles, P. J. The Determination of Point Groups from Imprecise Molecular Geometries. *J. Math. Chem.* **2022**, *60*, 161–171.

(25) Marston, C. C.; Balint-Kurti, G. G. The Fourier Grid Hamiltonian Method for Bound State Eigenvalues and Eigenfunctions. *J. Chem. Phys.* **1989**, *91*, 3571–3576.

(26) Webb, S. P.; Hammes-Schiffer, S. Fourier Grid Hamiltonian Multiconfigurational Self-Consistent-Field: A Method to Calculate Multidimensional Hydrogen Vibrational Wavefunctions. *J. Chem. Phys.* **2000**, *113*, 5214–5227.

(27) Rappoport, D.; Furche, F. Property-Optimized Gaussian Basis Sets for Molecular Response Calculations. *J. Chem. Phys.* **2010**, *133*, 134105.

(28) Haynes, W. M., Ed. *CRC Handbook of Chemistry and Physics*, 95th ed.; CRC Press, 2014.

(29) Liu, A.; Chow, M.; Wildman, A.; Frisch, M. J.; Hammes-Schiffer, S.; Li, X. Simultaneous Optimization of Nuclear–Electronic Orbitals. *J. Phys. Chem. A* **2022**, *126*, 7033–7039.

(30) Yoo, J. M.; Chow, M.; Paenurk, E.; Hammes-Schiffer, S. Reliable pK_a Prediction through Efficient Incorporation of Anharmonicity within the Nuclear–Electronic Orbital Framework. *J. Am. Chem. Soc.* **2025**, *147*, 36059–36065.

(31) Pavošević, F.; Culpitt, T.; Hammes-Schiffer, S. Multicomponent Coupled Cluster Singles and Doubles Theory within the Nuclear-Electronic Orbital Framework. *J. Chem. Theory Comput.* **2019**, *15*, 338–347.

(32) Fowler, D.; Brorsen, K. R. (T) Correction for Multicomponent Coupled-Cluster Theory for a Single Quantum Proton. *J. Chem. Theory Comput.* **2022**, *18*, 7298–7305.

(33) Goudy, R. J.; Pavošević, F.; Hammes-Schiffer, S. Triple Excitations in Nuclear–Electronic Orbital Coupled Cluster Theory for Multiple Quantum Protons. *J. Chem. Phys.* **2025**, *163*, 224119.

(34) Richardson, J. O. Ring-Polymer Instanton Theory. *Int. Rev. Phys. Chem.* **2018**, *37*, 171–216.

(35) Litman, Y. Tunneling and Zero-Point Energy Effects in Multidimensional Hydrogen Transfer Reactions: From Gas Phase to Adsorption on Metal Surfaces. Ph.D. thesis, 2020.
